# Histone deacetylases 1 and 2 restrain CD4^+^ cytotoxic T lymphocyte differentiation

**DOI:** 10.1172/jci.insight.133393

**Published:** 2020-02-27

**Authors:** Teresa Preglej, Patricia Hamminger, Maik Luu, Tanja Bulat, Liisa Andersen, Lisa Göschl, Valentina Stolz, Ramona Rica, Lisa Sandner, Darina Waltenberger, Roland Tschismarov, Thomas Faux, Thorina Boenke, Asta Laiho, Laura L. Elo, Shinya Sakaguchi, Günter Steiner, Thomas Decker, Barbara Bohle, Alexander Visekruna, Christoph Bock, Birgit Strobl, Christian Seiser, Nicole Boucheron, Wilfried Ellmeier

**Affiliations:** 1 Division of Immunobiology, Institute of Immunology, Center for Pathophysiology, Infectiology and Immunology, Medical University of Vienna, Vienna, Austria.; 2 Institute for Medical Microbiology and Hygiene, Philipps-University Marburg, Marburg, Germany.; 3 Institute of Animal Breeding and Genetics, Department of Biomedical Sciences, University of Veterinary Medicine Vienna, Vienna, Austria.; 4 Division of Rheumatology, Department of Internal Medicine III, Medical University of Vienna, Vienna, Austria.; 5 Max Perutz Labs, University of Vienna, Vienna, Austria.; 6 Medical Bioinformatics Centre, Turku Bioscience Centre, University of Turku and Åbo Akademi University, Turku, Finland.; 7 CeMM Research Center for Molecular Medicine of the Austrian Academy of Sciences, Vienna, Austria.; 8 Ludwig Boltzmann Institute for Arthritis and Rehabilitation, Vienna, Austria.; 9 Department of Pathophysiology and Allergy Research, Center for Pathophysiology, Infectiology and Immunology,; 10 Department of Laboratory Medicine, and; 11 Division of Cell and Developmental Biology, Center for Anatomy and Cell Biology, Medical University of Vienna, Vienna, Austria.

**Keywords:** Immunology, T cells

## Abstract

Some effector CD4^+^ T cell subsets display cytotoxic activity, thus breaking the functional dichotomy of CD4^+^ helper and CD8^+^ cytotoxic T lymphocytes. However, molecular mechanisms regulating CD4^+^ cytotoxic T lymphocyte (CD4^+^ CTL) differentiation are poorly understood. Here we show that levels of histone deacetylases 1 and 2 (HDAC1-HDAC2) are key determinants of CD4^+^ CTL differentiation. Deletions of both *Hdac1* and 1 *Hdac2* alleles (HDAC1^cKO^-HDAC2^HET^) in CD4^+^ T cells induced a T helper cytotoxic program that was controlled by IFN-γ–JAK1/2–STAT1 signaling. In vitro, activated HDAC1^cKO^-HDAC2^HET^ CD4^+^ T cells acquired cytolytic activity and displayed enrichment of gene signatures characteristic of effector CD8^+^ T cells and human CD4^+^ CTLs. In vivo, murine cytomegalovirus–infected HDAC1^cKO^-HDAC2^HET^ mice displayed a stronger induction of CD4^+^ CTL features compared with infected WT mice. Finally, murine and human CD4^+^ T cells treated with short-chain fatty acids, which are commensal-produced metabolites acting as HDAC inhibitors, upregulated CTL genes. Our data demonstrate that HDAC1-HDAC2 restrain CD4^+^ CTL differentiation. Thus, HDAC1-HDAC2 might be targets for the therapeutic induction of CD4^+^ CTLs.

## Abstract 2

Histone deacetylases 1 and 2 restrict naive CD4^+﻿^﻿ T cell differentiation into CD4^+﻿^﻿ cytotoxic T lymphocytes and might be targets for the therapeutic induction of CD4^+﻿^﻿ cytotoxic T lymphocytes.

## Graphical abstract

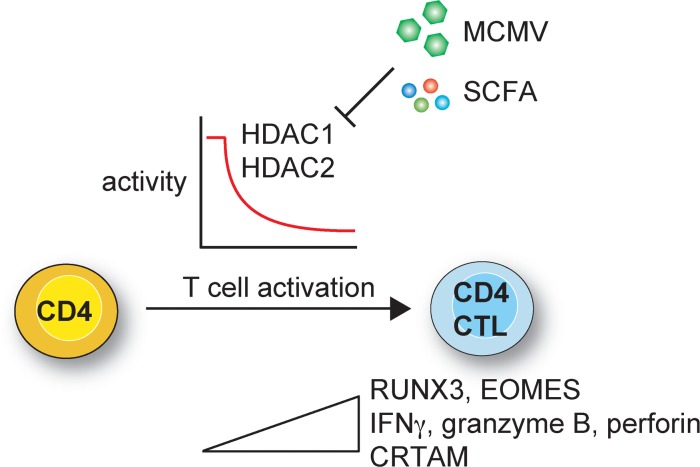

## Introduction

T lymphocytes are a key cell population of adaptive immunity. In addition to the classical functional T cell dichotomy of MHC class II–restricted CD4^+^ helper T cells (Th) and MHC class I–restricted cytotoxic CD8^+^ T cells, certain subsets of Th cells acquire cytotoxic activity. These CD4^+^ cytotoxic T lymphocytes (CD4^+^ CTLs) have been identified in patients with viral infections and contribute to antiviral immune responses (1, 2). CD4^+^ CTLs have also been implicated in antitumor immunity (3, 4) and have been linked with small intestinal inflammation in celiac disease (5). CD4^+^ CTLs are characterized by an upregulation of a cytotoxic program that includes CD8 lineage genes such as *Cd8a* (encoding CD8α), *Eomes* (encoding eomesodermin), *Tbx21* (encoding T-bet), *GzmB* (encoding granzyme B), *Prf1* (encoding perforin 1), and the degranulation marker *Lamp1* (encoding CD107a), and CD4^+^ CTLs also produce high levels of IFN-γ (encoded by *Ifng*) (6–8). Recent data indicate that CD4^+^ CTLs are derived from activated CD4^+^ T cells that express MHC class I–related T cell–associated molecule (CRTAM) (9). However, in contrast to the well-defined regulatory networks leading to the differentiation of Th1, Th2, and Th17 cells, the pathways that drive the differentiation of naive CD4^+^ T cells into CD4 CTLs are only poorly understood.

During the differentiation of naive CD4^+^ T cells into effector T cells, cell fate decisions into various Th subsets are made, and Th cell lineage-specific gene expression patterns are established and maintained. Epigenetic mechanisms, such as histone and DNA modifications, play a crucial role in these processes. Among these, modification of core histones by reversible lysine acetylation is controlled by histone acetyltransferases (HATs) and histone deacetylases (HDACs), which are “classically” considered as transcriptional coactivators and corepressors, respectively. However, HDACs are also recruited to active gene loci and might, potentially with HATs, act context dependently as modulators of gene transcription. Moreover, many nonhistone targets have been emerging, and HATs/HDACs function beyond the epigenetic control of gene expression (10–12). To date, 18 members of the HDAC family (many of which are expressed in the T cell lineage) that are grouped into 4 classes have been identified (13). We have recently generated mice with a T cell–specific deletion of the class I histone deacetylases HDAC1 and HDAC2, which resulted in MHC class II–restricted CD4^+^CD8αβ^+^ T cells that, upon activation, initiate the upregulation of a Runx3/CBFβ-dependent CD8 effector T cell–like program (14, 15). This observation indicates that CD4 lineage integrity is regulated by HDAC1/HDAC2 and raises the exciting prospect that HDAC1 and HDAC2 may be part of the regulatory network controlling CD4^+^ CTL differentiation. However, T cell numbers in HDAC1-2 conditional double-knockout (HDAC1-2^cDKO^) mice are substantially compromised in vivo, and HDAC1-HDAC2 double-deficient CD4^+^ T cells undergo apoptosis upon activation (14), which precluded their in-depth analysis and the dissection of the individual contributions of HDAC1 and HDAC2.

To test whether activated CD4^+^ T cells lacking HDAC1 and HDAC2 acquire CD4^+^ CTL characteristics, including gene signatures and cytolytic activity, and whether CD4^+^ CTL induction in response to viral infection is regulated by HDAC1 and HDAC2, we generated mice with a T cell–specific combinatorial deletion of 3 of the 4 *Hdac1* and *Hdac2* alleles. Moreover, we analyzed human CD4^+^ T cells treated with the class I HDAC inhibitor entinostat or with short-chain fatty acids, which are commensal-produced metabolites that have HDAC-inhibitory activity. Our study indicates that HDAC1 and HDAC2 are key regulators of CD4^+^ CTL differentiation.

## Results

### HDAC1/HDAC2 dosage–dependent effects on CTL lineage gene induction in CD4^+^ T cells.

**Figure 1:**
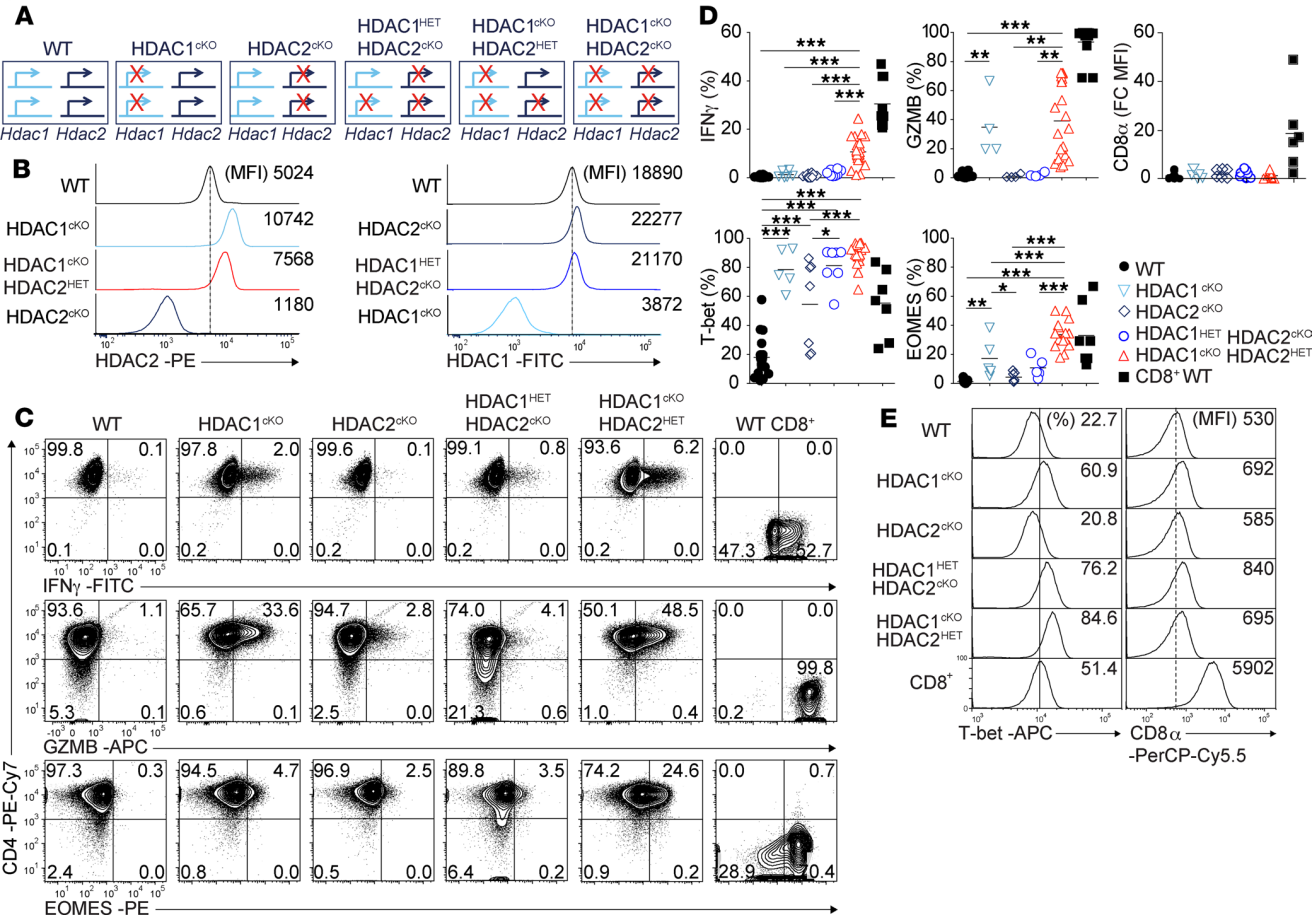
HDAC1/HDAC2 dosage–dependent upregulation of CD8 lineage factors in CD4^+^ T cells. (**A**) Overview of the different mouse strains used for the experiments. The crossed arrows indicate deleted *Hdac1* and *Hdac2* alleles. (**B**) Histograms showing HDAC2 and HDAC1 expression levels in TCRβ^+^CD4^+^ splenocytes isolated from WT, HDAC1^cKO^, HDAC2^cKO^, HDAC1^HET^-HDAC2^cKO^, and HDAC1^cKO^-HDAC2^HET^ mice. (**C**) Flow cytometry analyses showing CD4, IFN-γ, granzyme B (GzmB), and EOMES expression in WT, HDAC1^cKO^, HDAC2^cKO^, HDAC1^HET^-HDAC2^cKO^, and HDAC1^cKO^-HDAC2^HET^ CD4^+^ T cells and WT CD8^+^ T cells activated with anti-CD3 and anti-CD28 for 3 days in the presence of IL-2. (**D**) Summary diagrams showing the percentages of IFN-γ^+^, granzyme B^+^, T-bet^+^, and EOMES^+^ cells of the indicated genotype as described in **C** and **D**. For CD8α^+^, WT MFI levels were set as 1, and relative MFI levels in HDAC1^cKO^, HDAC2^cKO^, HDAC1^HET^-HDAC2^cKO^, and HDAC1^cKO^-HDAC2^HET^ CD4^+^ and WT CD8^+^ T cells are shown. Each symbol indicates 1 independent biological sample. Horizontal bars indicate the mean. *P < 0.05, **P < 0.01, and ***P < 0.001, 1-way ANOVA analysis followed by Tukey’s multiple-comparisons test (CD8^+^ WT is shown as control and was not included in the statistical analysis). (**E**) Histograms showing T-bet and CD8 expression in WT, HDAC1^cKO^, HDAC2^cKO^, HDAC1^HET^-HDAC2^cKO^, and HDAC1^cKO^-HDAC2^HET^ CD4^+^ and WT CD8^+^ T cells activated as described in **C**. (**B**, **C**, and **E**) Numbers indicate the percentage of cells in the respective quadrants or gates or, as indicated, the MFI. (**B** and **E**) The dotted vertical lines indicate the peak of the WT histogram (for MFI), while the vertical solid line indicates the gating region for the percentage of cells. Data are representative (**B**, **C**, and **E**) or show a summary (**D**) of at least 7 (**B**) or 6 (**C**, **D**, and **E**) mice that were analyzed in at least 3 (**B**) or 4 (**C**, **D**, and **E**) independent experiments.

HDAC1-HDAC2 double-deficient (HDAC1-2^cDKO^) CD4^+^ T cells undergo apoptosis upon activation (14). We hypothesized that HDAC1 or HDAC2 expression levels above a certain threshold level in the absence of the corresponding other member might be sufficient to rescue CD4^+^ T cells from apoptosis and thus might offer the possibility to study their role in regulating CD4^+^ CTLs’ induction. To test this hypothesis, we generated mice that express either only 1 *Hdac2* allele (*Hdac1*^fl/fl^ *Hdac2*^fl/+^ *Cd4*-Cre; HDAC1^cKO^-HDAC2^HET^) or 1 *Hdac1* allele (HDAC1^HET^-HDAC2^cKO^) (Figure 1A). Of note, HDAC1^cKO^ and HDAC2^cKO^ CD4^+^ T cells upregulated HDAC2 or HDAC1 (Figure 1B), respectively, as previously reported (14, 16). The analysis of HDAC2 expression in ex vivo–isolated HDAC1^cKO^-HDAC2^HET^ CD4^+^ T cells revealed lower HDAC2 levels in comparison with HDAC1^cKO^ CD4^+^ T cells (Figure 1B). A similar reduction in HDAC1 expression levels in comparison with HDAC2^cKO^ CD4^+^ T cells was observed ex vivo in HDAC1^HET^-HDAC2^cKO^ CD4^+^ T cells (Figure 1B). To study the effect of lowered HDAC1 or HDAC2 expression in more detail, naive CD4^+^ T cells from HDAC1^cKO^, HDAC2^cKO^, HDAC1^HET^-HDAC2^cKO^, and HDAC1^cKO^-HDAC2^HET^ mice were sorted and activated with anti-CD3/anti-CD28 under nonpolarizing (“Th0”) conditions for 3 days. In contrast to HDAC1-2^cDKO^ CD4^+^ T cells (14), “adding” 1 *Hdac2* allele back to HDAC1/HDAC2-deficient CD4^+^ T cells (HDAC1^cKO^-HDAC2^HET^) led to a similar proliferation and survival upon activation in vitro as observed for activated WT CD4^+^ T cells (Supplemental Figure 1, A and B; supplemental material available online with this article; https://doi.org/10.1172/jci.insight.133393DS1). As previously reported (16, 17), HDAC1^cKO^ CD4^+^ T cells upregulated IFN-γ (Figure 1C). Moreover, we observed that activated HDAC1^cKO^ CD4^+^ T cells also expressed enhanced levels of EOMES, granzyme B, and T-bet in comparison with activated WT CD4^+^ T cells (Figure 1, C–E), indicating upregulation of several genes characteristic for Th cytotoxicity and CTLs. The expression of some of these proteins was also increased in HDAC2^cKO^ CD4^+^ T cells but to a lower degree in comparison with HDAC1^cKO^ CD4^+^ T cells. Deletion of 1 *Hdac1* allele in the absence of HDAC2 (HDAC1^HET^-HDAC2^cKO^) led to an increase in CTL lineage gene expression in comparison with HDAC2^cKO^ cells (Figure 1, C–E). Moreover, the deletion of 1 *Hdac2* allele on top of HDAC1 deficiency (HDAC1^cKO^-HDAC2^HET^) led to the highest upregulation of CTL lineage genes (Figure 1, C–E). A similar graded upregulation of CTL lineage genes was also observed in Th1 cells (Supplemental Figure 1, C–E). Together, these data show an *Hdac1* and *Hdac2* gene dosage–dependent upregulation of Th cytotoxic genes, with HDAC1 activity being the most essential for the repression of CTL lineage genes. In contrast to HDAC1-2^cKO^ CD4^+^ T cells that display a strong upregulation of CD8α expression (14), CD8α protein expression was not detected (or only at very low levels) in activated CD4^+^ T cells of the various genotypes (Figure 1, D and E). These data indicate an *Hdac1* and *Hdac2* gene dosage–dependent induction of CTL features in CD4^+^ T cells. Because HDAC1^cKO^-HDAC2^HET^ CD4^+^ T cells displayed the strongest phenotype, WT and HDAC1^cKO^-HDAC2^HET^ CD4^+^ T cells and mice were used for subsequent experiments.

### Transcriptional changes in HDAC1^cKO^-HDAC2^HET^ CD4^+^ T cells.

**Figure 2:**
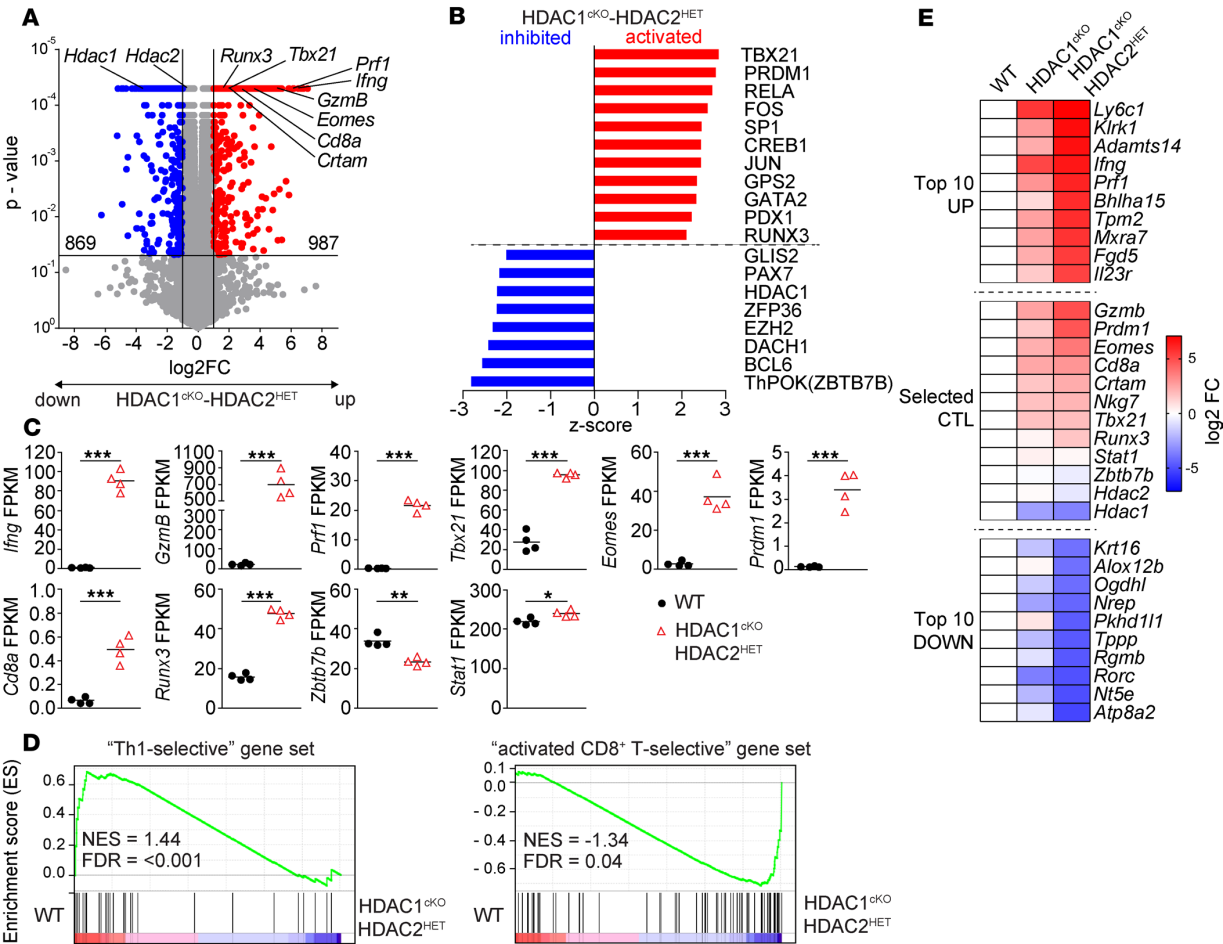
Induction of a CD4^+^ CTL signature in activated HDAC1^cKO^-HDAC2^HET^ CD4^+^ T cells. Naive WT and HDAC1^cKO^-HDAC2^HET^ CD4^+^ T cells were activated with anti-CD3/anti-CD28 for 3 days in the presence of IL-2. RNA was isolated from sorted viable cells and subjected to RNA-Seq. On the same day 4 independent WT and HDAC1^cKO^-HDAC2^HET^ CD4^+^ T cell batches were prepared. (**A**) Volcano plot depicts a comparison of global gene expression profiles between activated WT and HDAC1^cKO^-HDAC2^HET^ CD4^+^ T cells. 987 and 869 genes were up- and downregulated, respectively, in HDAC1^cKO^-HDAC2^HET^ CD4^+^ T cells (FC ≥ 2; FDR ≤ 0.05). (**B**) Diagram showing top hits of upstream transcriptional regulators (–2 ≥ *Z* score ≥ 2; *P* ≤ 0.05), as revealed by Ingenuity Pathway Analysis (QIAGEN Inc.), that are predicted to be “activated” or “inhibited” in HDAC1^cKO^-HDAC2^HET^ CD4^+^ T cells. The *x* axis indicates the *Z* score. (**C**) Summary diagrams depict the expression (values shown as fragments per kilobase of transcript per million mapped reads; FPKM) of the indicated genes in activated WT and HDAC1^cKO^-HDAC2^HET^ CD4^+^ T cells as determined by RNA-Seq. Each symbol indicates 1 biological sample. Horizontal bars indicate the mean. *P < 0.05, **P < 0.01, and ***P < 0.001 (unpaired 2-tailed Student’s *t* test). (**D**) Gene set enrichment analysis (GSEA) plots of Th1-specific and CD8 lineage–specific gene sets (containing 169 and 477 genes, respectively) in activated HDAC1^cKO^-HDAC2^HET^ CD4^+^ T cells relative to activated WT CD4^+^ T cells. The barcodes indicate the location of the members of the gene set in the ranked list of all genes. NES, normalized enrichment score in WT as compared with HDAC1^cKO^-HDAC2^HET^ population. (**E**) Heatmap showing fold change (FC) differences of the top 10 up- and downregulated genes (excluding noncoding RNAs) based on log_2_ FC as well as of selected CTL genes between activated WT and HDAC1^cKO^-HDAC2^HET^ CD4^+^ T cells (activated as described in **A**). The second lane shows FC differences of these genes between activated WT and HDAC1^cKO^ CD4^+^ T cells (anti-CD3/anti-CD28 for 3 days, restimulated with anti-CD3 for 12 hours as previously described) (17).

To assess changes in HDAC1^cKO^-HDAC2^HET^ CD4^+^ T cells in greater detail, we determined the transcriptome of anti-CD3/anti-CD28 activated WT and HDAC1^cKO^-HDAC2^HET^ CD4^+^ T cells using RNA-sequencing (RNA-Seq) approaches. We found 987 genes upregulated and 869 genes downregulated in HDAC1^cKO^-HDAC2^HET^ CD4^+^ T cells in comparison with WT cells (Figure 2A). Pathway analysis identified a high number of transcription factors as top upstream regulators (defined by a *Z* score either ≤ –2 or ≥ 2), among them activation of T-bet– and RUNX3*-*dependent programs and inhibition of ThPOK-regulated (also known as ZBTB7B, encoded by *Zbtb7b*) pathways (Figure 2B). Further, genes associated with a Th cytotoxic program were upregulated, such as *Ifng*, *GzmB*, *Prf1*, *Tbx21*, *Runx3*, and *Eomes*, while CD4 lineage genes, such as *Zbtb7b*, were downregulated (Figure 2C). We also observed upregulation of *Cd8a* gene expression, although at very low expression levels (FPKM < 0.5) (Figure 2C), correlating with the undetectable or low-level CD8α protein expression determined by flow cytometry (Figure 1D). Moreover, in agreement with a study showing opposing development of CD4^+^ cytotoxic T cells and T follicular helper cells (18), BCL6 signatures were downregulated, and Blimp-1 (encoded by *Prdm1*) signatures (Figure 2B) as well as *Prdm1* expression (Figure 2C) were upregulated. To obtain an understanding whether expression signatures in HDAC1^cKO^-HDAC2^HET^ CD4^+^ T cells represent Th1-like or CTL lineage patterns, we performed gene set enrichment analyses (GSEAs). We compared the differentially expressed gene list with “Th1-selective” gene sets and “activated CTL-selective” gene sets that have been generated from a comparison of published Th1 and activated CD8^+^ T cell (CTL) microarray data sets (19) (Supplemental Table 1). Although the “Th1-selective” gene set was underrepresented in HDAC1^cKO^-HDAC2^HET^ CD4^+^ T cells, “activated CTL-selective” genes were enriched in HDAC1^cKO^-HDAC2^HET^ CD4^+^ T cells (Figure 2D). Additional GSEAs with annotated gene sets from the Molecular Signatures Database (20) revealed an up- or downregulation of other signatures in HDAC1^cKO^-HDAC2^HET^ CD4^+^ T cells, although the normalized enrichment scores were the highest in “Th1-selective” and “activated CTL-selective“ gene sets (Supplemental Table 2). Together, the transcriptome analysis suggests the induction of a CTL lineage program in HDAC1^cKO^-HDAC2^HET^ CD4^+^ T cells. Of note, fold change (FC) expression differences of the top 10 most up- and downregulated genes as well as selected Th cytotoxic lineage genes between activated HDAC1^cKO^-HDAC2^HET^ and WT CD4^+^ T cells were higher compared with FC expression differences between HDAC1^cKO^ and WT CD4^+^ T cells (17) (Figure 2E). This indicates an *Hdac1* and *Hdac2* gene dosage–dependent effect on gene expression levels between HDAC1^cKO^ and HDAC1^cKO^-HDAC2^HET^ CD4^+^ T cells.

### HDAC1^cKO^-HDAC2^HET^ CD4^+^ T cells display cytotoxic activity in vitro.

**Figure 3:**
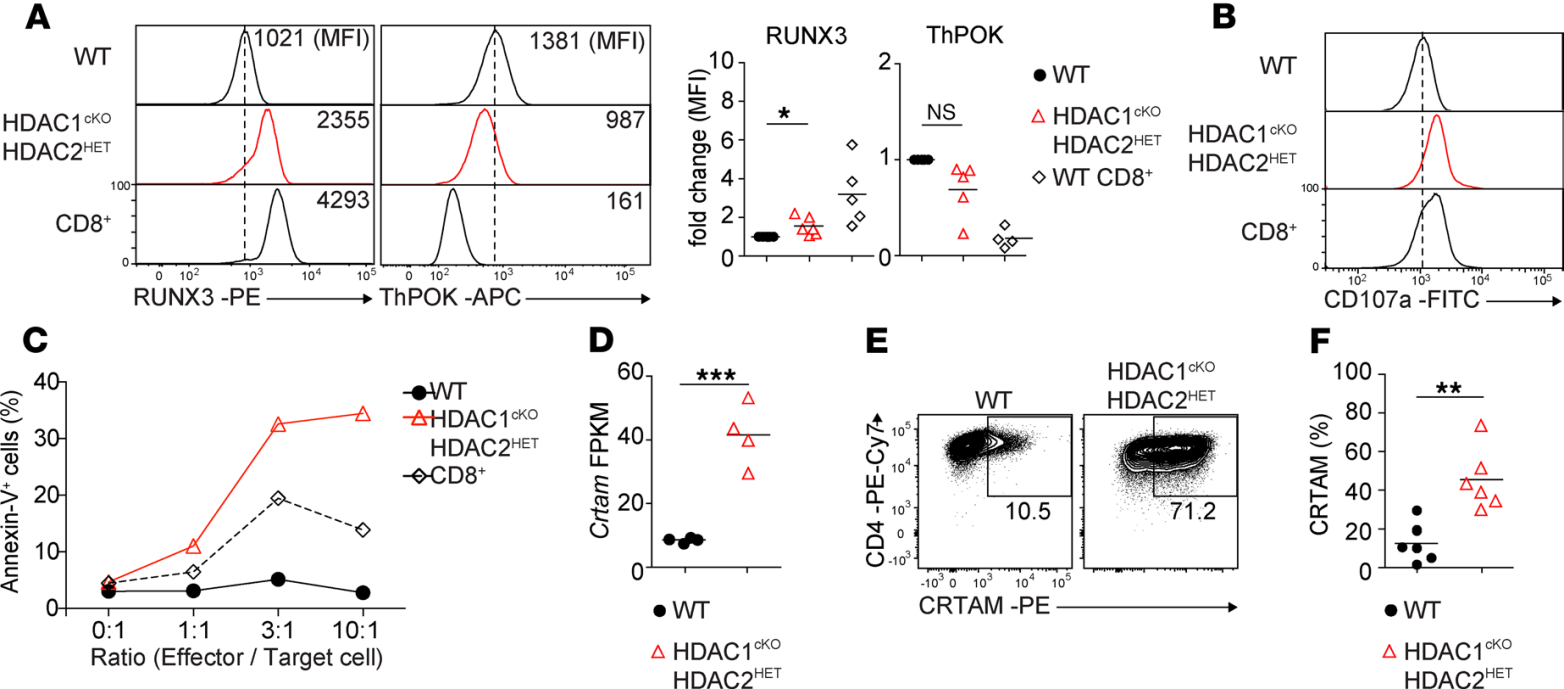
HDAC1^cKO^-HDAC2^HET^ CD4^+^ T cells display CTL activity. (**A**) Histograms show RUNX3 and ThPOK expression in naive WT and HDAC1^cKO^-HDAC2^HET^ CD4^+^ and WT CD8^+^ T cells activated with anti-CD3/anti-CD28 for 3 days in the presence of IL-2. Diagrams depict the summary of the MFI of RUNX3 and ThPOK expression levels of all independent experiments. WT MFI levels were set as 1, and relative MFI levels in HDAC1^cKO^-HDAC2^HET^ CD4^+^ and WT CD8^+^ T cells are shown. Each symbol indicates 1 mouse. Horizontal bars indicate the mean. (**B**) Histograms depict CD107a expression on naive WT and HDAC1^cKO^-HDAC2^HET^ CD4^+^ and WT CD8^+^ T cells activated as described in **A**. (**C**) Redirected cytotoxicity assay using WT and HDAC1^cKO^-HDAC2^HET^ CD4^+^ and WT CD8^+^ T cells. Effector cells were prepared by activating naive CD4^+^ and CD8^+^ T cells with anti-CD3/anti-CD28 for 3 days in the presence of IL-2. On day 3 activated cells were cocultured with P815 target cells at the indicated ratios in the presence of soluble anti-CD3. Target cells were stained 4 hours later with 7-AAD/annexin V and quantified by flow cytometry. Percentage of annexin V^+^ target cells and the effector/target cell ratio are indicated. (**D**) Summary diagram indicates FPKM values of *Crtam* expression in activated WT and HDAC1^cKO^-HDAC2^HET^ CD4^+^ T cells as determined by RNA-Seq. (**E**) Contour plots show CRTAM expression on WT and HDAC1^cKO^-HDAC2^HET^ CD4^+^ T cells activated as described in **A**. (**F**) Summary of experiments described in **E**. Diagram depicts the percentages of activated CD4^+^ T cells expressing CRTAM. (**A**, **D**, and **F**) Each symbol indicates 1 biological sample. Horizontal bars indicate the mean. *P < 0.05, **P < 0.01, and ***P < 0.001 (unpaired 2-tailed Student’s *t* test). (**A**, **B**, and **E**) Numbers indicate the MFI (**A**) or the percentage of cells in the respective regions (**E**). (**A** and **B**) The dotted vertical lines indicate the peak of the WT histogram (for MFI). Data are representative of at least 3 (**A–C**) or 4 (**D–F**) mice that were analyzed in at least 5 (**A**), 2 (**E** and **F**), or 3 (**B** and **C**) independent experiments or 1 (**D**) experiment.

CD4^+^ T cells can be reprogrammed to develop into intestinal intraepithelial CD4^+^ CTLs (21, 22). This reprogramming is accompanied by a downregulation of the CD4 lineage commitment factor ThPOK and a corresponding upregulation of RUNX3, a key factor for the development of CD8 lineage T cells (21, 22). Therefore, we analyzed the expression of RUNX3 and ThPOK in WT and HDAC1^cKO^-HDAC2^HET^ CD4^+^ T cells cultured for 3 days under nonpolarizing conditions. In agreement with the RNA-Seq data (Figure 2, A and C), RUNX3 protein expression was upregulated, while ThPOK protein expression was downregulated in activated HDAC1^cKO^-HDAC2^HET^ CD4^+^ T cells compared with WT CD4^+^ T cells (Figure 3A). This indicates similar changes in the expression of these key transcription factors in HDAC1^cKO^-HDAC2^HET^ CD4^+^ T cells as observed during the conversion of CD4^+^ T cells into CD4^+^ CTLs (22). Further, HDAC1^cKO^-HDAC2^HET^ CD4^+^ T cells also upregulated the degranulation marker CD107a (Figure 3B), which is known to correlate with the cytolytic potential and cytokine expression of CD8^+^ T cells (23). In vitro redirected cytotoxicity assays demonstrated that activated HDAC1^cKO^-HDAC2^HET^ CD4^+^ T cells displayed CTL activity toward P815 target cells as revealed by annexin V staining, indicating that reduced HDAC1 and HDAC2 activity led to the generation of CD4^+^ T cells with cytotoxic activity (Figure 3C). Notably, CRTAM was highly upregulated at the mRNA as well as the protein level in activated HDAC1^cKO^-HDAC2^HET^ CD4^+^ T cells (Figure 3, D–F). Because CRTAM is critical to direct CD4^+^ CTLs’ differentiation (9), these data demonstrate that in vitro activated HDAC1^cKO^-HDAC2^HET^ CD4^+^ T cells displayed another characteristic feature of CD4^+^ CTLs. Together, these results indicate that activated HDAC1^cKO^-HDAC2^HET^ CD4^+^ T cells share key features and gene signatures of mouse CTLs and that HDAC1-HDAC2 regulate the induction of CD4^+^ CTLs.

### IFN-γ–JAK1/2–STAT1 signaling is required for CTL features in HDAC1^cKO^-HDAC2^HET^ CD4^+^ T cells.

**Figure 4:**
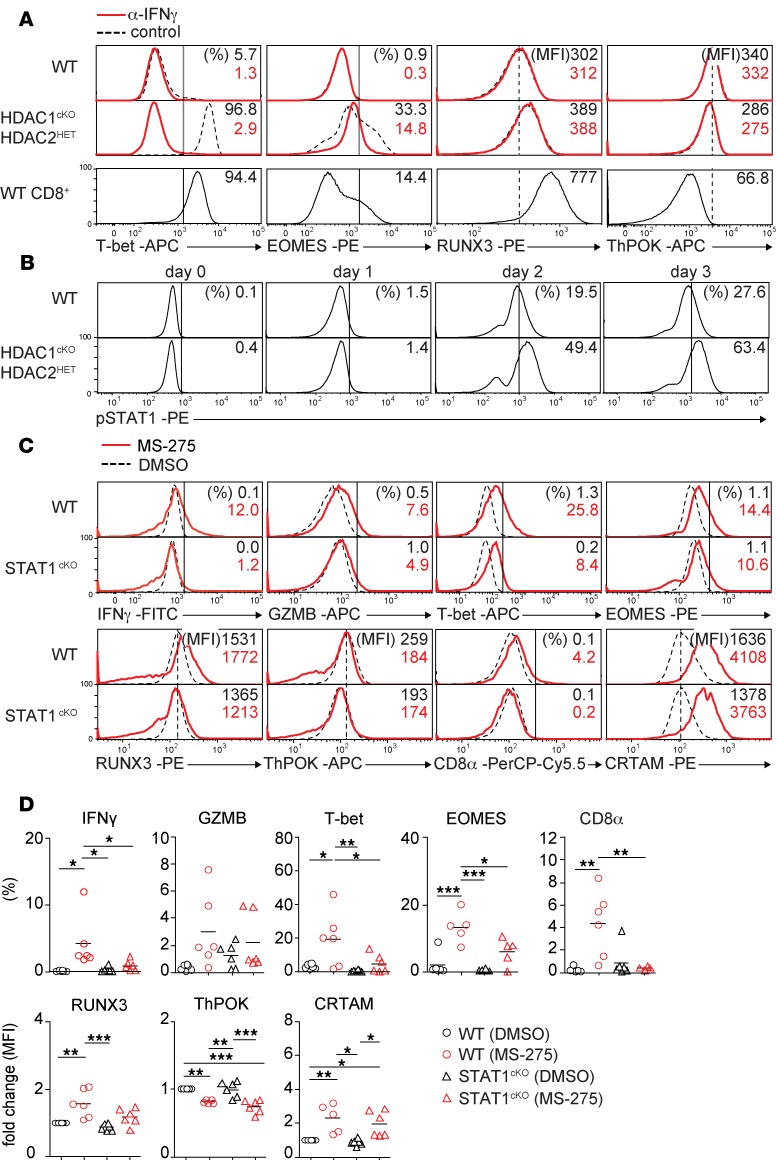
An IFN-γ–JAK1/2–STAT1 signaling pathway is required for CD4^+^ CTL features in HDAC1^cKO^-HDAC2^HET^ CD4^+^ T cells. (**A**) Histogram overlays depict T-bet, EOMES, RUNX3, and ThPOK expression in naive WT (upper panel), HDAC1^cKO^-HDAC2^HET^ CD4^+^ (middle panel) and WT CD8^+^ T cells (lower panel) activated with anti-CD3/anti-CD28 for 3 days in the presence (solid red line) or absence (control, dotted black line) of IFN-γ–blocking antibodies (α–IFN-γ). (**B**) Histograms depict p-STAT1 levels in naive WT and HDAC1^cKO^-HDAC2^HET^ CD4^+^ T cells activated with anti-CD3/anti-CD28 in the presence of IL-2 and analyzed by flow cytometry at the indicated time points. (**C**) Histograms depict IFN-γ, granzyme B, T-bet, EOMES, RUNX3, ThPOK, CD8, and CRTAM expression in naive WT and STAT1^cKO^ CD4^+^ T cells activated with anti-CD3/anti-CD28 for 3 days. During the last 24 hours before analysis, DMSO (control, dotted black line) and the HDACi MS-275 (solid red line) were added. (**D**) Summary of experiments described in **C**. Diagrams depict the percentages of CD4^+^ T cells expressing the indicated cytokines/transcription factors; or WT (DMSO) MFI levels were set as 1, and relative MFI levels in WT (MS-275) and HDAC1^cKO^-HDAC2^HET^ (DMSO/MS-275) CD4^+^ T cells are shown. Each symbol indicates 1 independent biological sample. Horizontal bars indicate the mean. *P < 0.05, **P < 0.01, and ***P < 0.001 (1-way ANOVA analysis followed by Tukey’s multiple-comparisons test). (**A–C**) Numbers indicate the percentage of cells in the respective quadrants and gates or, as indicated, the MFI. The dotted vertical lines indicate the peak of the WT histogram (for MFI), while the vertical solid lines indicate the gating region for the percentage of cells. Data are representative of at least 5 (**A**) or 4 (**B–D**) mice that were analyzed in at least 3 (**A**, **C**, and **D**) or 2 (**B**) independent experiments.

It has been shown that CD4^+^ CTLs produce IFN-γ and can develop from Th0 cells (24, 25), although other Th subsets also give rise to CD4^+^ CTLs (26–28). Because IFN-γ expression was increased in activated HDAC1^cKO^-HDAC2^HET^ CD4^+^ T cells (Figure 1C), we tested whether the induction of Th cytotoxic features is a consequence of elevated IFN-γ levels. Therefore, we activated WT and HDAC1^cKO^-HDAC2^HET^ CD4^+^ T cells with anti-CD3/anti-CD28 in the absence or presence of a neutralizing anti–IFN-γ antibody. Cytotoxic genes were induced in HDAC1^cKO^-HDAC2^HET^ CD4^+^ T cells in comparison with WT CD4^+^ T cells in control Th0 cultures; however, adding neutralizing anti–IFN-γ antibodies to HDAC1^cKO^-HDAC2^HET^ CD4^+^ T cells resulted in decreased expression of IFN-γ, granzyme B, T-bet, and EOMES (Figure 4A and Supplemental Figure 2, A and B) while RUNX3 and ThPOK expression was not changed by neutralizing IFN-γ (Figure 4A and Supplemental Figure 2B). The addition of anti–IFN-γ antibody to WT CD4^+^ T cells did not have an effect on the expression of these molecules (Figure 4A and Supplemental Figure 2B). Of note, exogenous high-level IFN-γ did not induce CD4^+^ CTL features in activated WT CD4^+^ T cells (Supplemental Figure 2C). Moreover, HDAC1^cKO^-HDAC2^HET^ CD4^+^ T cells did not produce soluble factors or express surface molecules that lead to the induction of Th cytotoxic features, since WT CD4^+^ T cells (CD45.1) cocultured with HDAC1^cKO^-HDAC2^HET^ CD4^+^ T cells (CD45.2^+^) (Supplemental Figure 3A) did not upregulate IFN-γ and granzyme B (Supplemental Figure 3B) and showed a reduced upregulation of T-bet and EOMES compared with the cocultured HDAC1^cKO^-HDAC2^HET^ CD4^+^ T cells (CD45.2^+^) (Supplemental Figure 3B). Further, WT CD4^+^ T cells had neither downregulated ThPOK nor upregulated RUNX3 expression in the presence of HDAC1^cKO^-HDAC2^HET^ CD4^+^ T cells (CD45.2^+^) (Supplemental Figure 3C). Together, these data indicate that IFN-γ signaling is key to but not sufficient for the induction of CTL features in HDAC1^cKO^-HDAC2^HET^ CD4^+^ T cells and that cell-autonomous, T cell–intrinsic mechanisms control the upregulation of the Th cytotoxic program in HDAC1^cKO^-HDAC2^HET^ CD4^+^ T cells.

T cell receptor (TCR) triggering together with IFN-γ stimulation leads to activation of STAT1 via JAK1/2 (29, 30). In a previous study we demonstrated that HDAC1 is a crucial negative regulator of STAT1 activation in CD4^+^ T cells, since activated HDAC1-deficient CD4^+^ T cells display increased levels of phospho-STAT1 (p-STAT1) (17). Intracellular staining also revealed a strong induction of p-STAT1 in HDAC1^cKO^-HDAC2^HET^ CD4^+^ T cells (Figure 4B) but not of *Stat1* gene expression (Figure 2C), while STAT5 phosphorylation was not affected (Supplemental Figure 4A). Ruxolitinib, a JAK1/2 inhibitor (31), abolished the induction of IFN-γ, granzyme B, and EOMES and reduced the induction of T-bet and RUNX3 (Supplemental Figure 4B). In contrast, the JAK3 inhibitor tofacitinib (32) did not block the induction of IFN-γ, T-bet, and EOMES and the downregulation of ThPOK, although the expression of granzyme B and RUNX3 were slightly reduced in comparison with untreated controls (Supplemental Figure 4C). This suggests a requirement for JAK1/2, but not for JAK3, signaling pathways during the induction of Th cytotoxic features in HDAC1^cKO^-HDAC2^HET^ CD4^+^ T cells. To directly address the role of STAT1 for CD4^+^ CTL induction, we treated WT and STAT1-deficient CD4^+^ T cells with the class I HDAC inhibitor (HDACi) MS-275 (entinostat). We previously showed that treatment of CD4^+^ T cells with MS-275 leads to the induction of CD8 lineage genes, such as CD8α, IFN-γ, granzyme B, T-bet, EOMES, and RUNX3 (14). In agreement with this study, we observed upregulation of these proteins in MS-275–treated activated WT CD4^+^ T cells (Figure 4, C and D). Moreover, MS-275 treatment led to upregulation of CRTAM and downregulation of ThPOK expression (Figure 4, C and D). In contrast, STAT1 deficiency in CD4^+^ T cells reduced the upregulation of these factors in response to MS-275 with the exception of CRTAM, which remained strongly upregulated in comparison with DMSO-treated controls, and ThPOK, which was still downregulated (Figure 4, C and D). Together, these data uncover IFN-γ–JAK1/2–STAT1 signaling as a key regulatory pathway in class I HDAC–controlled conversion of CD4^+^ T cells into CD4^+^ CTLs.

### The upregulation of CD4^+^ CTL features is partially independent of ThPOK downregulation.

**Figure 5:**
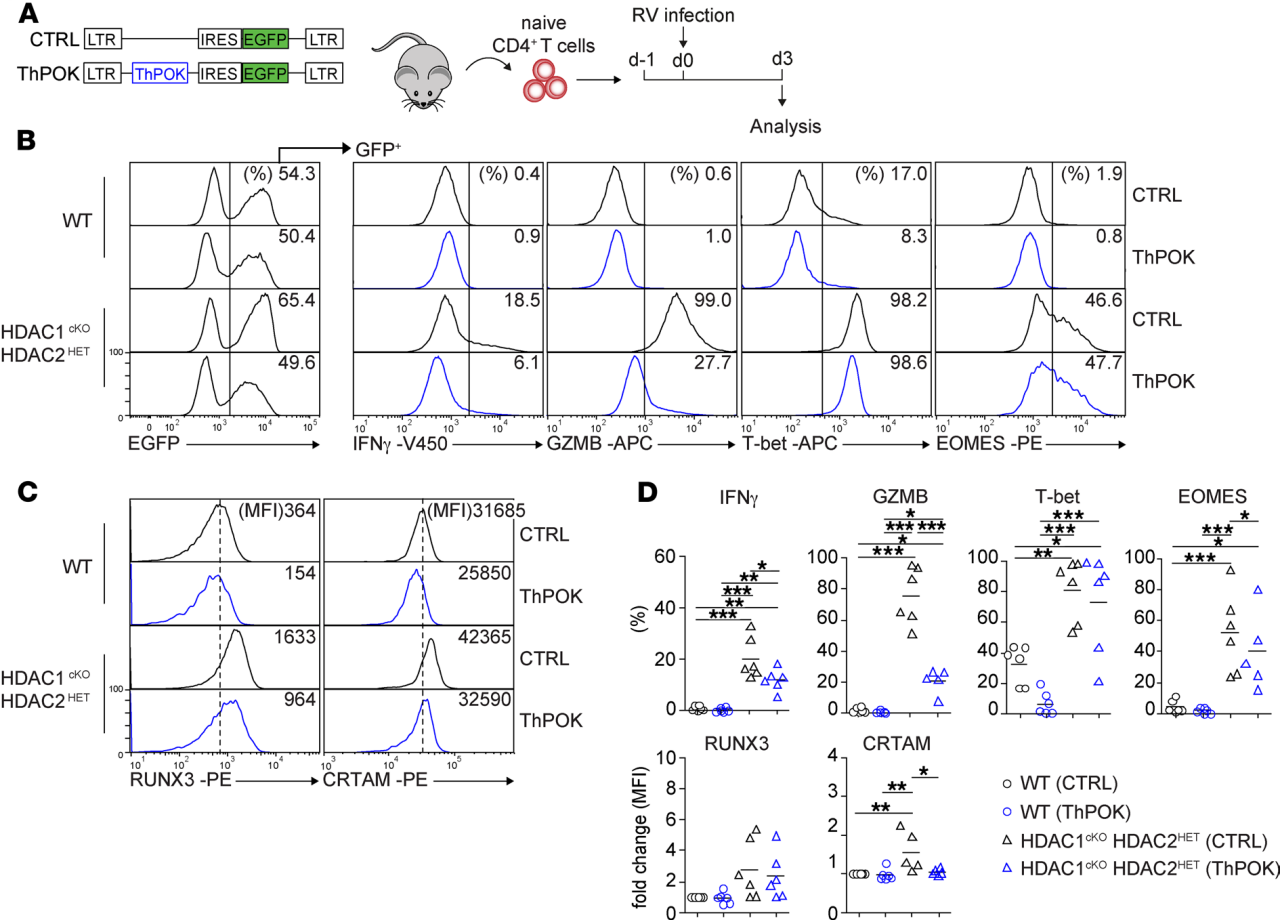
Enforced expression of ThPOK partially blocks the upregulation of Th cytotoxic features in HDAC1^cKO^-HDAC2^HET^ CD4^+^ T cells. (**A**) Experimental strategy: naive WT and HDAC1^cKO^-HDAC2^HET^ CD4^+^ T cells were activated with anti-CD3/anti-CD28 in the presence of IL-2 (d-1); 24 hours later (d0) were transduced with “empty” control-EGFP (CTRL) and ThPOK-EGFP retroviral vectors, respectively; and were further cultured for 3 days. (**B**) Histograms at the left depict EGFP^+^ expression in transduced CD4^+^ T cells. The other histograms depict IFN-γ, granzyme B, T-bet, and EOMES expression in EGFP^+^ WT and HDAC1^cKO^-HDAC2^HET^ CD4^+^ T cells transduced with either CTRL or ThPOK retroviral vectors. (**C**) Histograms depict RUNX3 and CRTAM expression in EGFP^+^ WT and HDAC1^cKO^-HDAC2^HET^ CD4^+^ T cells transduced with CTRL or ThPOK vector. (**D**) Summary of experiments described in **B** and **C**. Diagrams depict ether the percentages of CD4^+^ T cells expressing the indicated cytokines/transcription factors; or WT (CTRL) MFI levels were set as 1, and relative MFI levels in WT (ThPOK) and HDAC1^cKO^-HDAC2^HET^ (CTRL/ThPOK) CD4^+^ T cells are shown. Each symbol indicates 1 independent biological sample. Horizontal bars indicate the mean. *P < 0.05, **P < 0.01, and ***P < 0.001 (1-way ANOVA analysis followed by Tukey’s multiple-comparisons test). (**B** and **C**) Numbers indicate the percentage of cells in the respective regions or, as indicated, the MFI. The dotted vertical lines indicate the peak of the WT histogram (for MFI), while the vertical solid lines indicate the gating region for the percentage of cells. (**B–D**) Data are representative of at least 4 independent biological samples that were analyzed in at least 3 independent experiments.

ThPOK is a key regulator of mature CD4^+^ T cells’ transdifferentiation into CD8 lineage T cells (19). Next, we investigated whether the observed downregulation of ThPOK expression is essential for the induction of the CTL program in HDAC1^cKO^-HDAC2^HET^ CD4^+^ T cells. Therefore, we retrovirally expressed ThPOK (using an internal ribosome entry site–EGFP [IRES-EGFP] cassette to track transduced cells) in WT and HDAC1^cKO^-HDAC2^HET^ CD4^+^ T cells (Figure 5A). Enforced expression of ThPOK in HDAC1^cKO^-HDAC2^HET^ CD4^+^ T cells impaired the induction of IFN-γ, granzyme B, and RUNX3 expression in comparison with control retroviral vector–transduced (CTRL-transduced) HDAC1^cKO^-HDAC2^HET^ CD4^+^ T cells (Figure 5, B–D), although the expression levels of granzyme B and RUNX3 were still higher in comparison with WT CD4^+^ T cells (Figure 5, B–D). In contrast, the expression of EOMES and T-bet were not affected upon overexpression of ThPOK (Figure 5, B–D). Of note, enforced expression of ThPOK in WT CD4^+^ T cells resulted in a decreased induction of RUNX3 and T-bet expression. Finally, enforced ThPOK expression impaired the upregulation of CRTAM in HDAC1^cKO^-HDAC2^HET^ CD4^+^ T cells, suggesting that the downmodulation of ThPOK precedes the upregulation of CRTAM (Figure 5, C and D). Together, these data indicate that ThPOK overexpression is not sufficient to fully repress the induction of a cytotoxic program in HDAC1^cKO^-HDAC2^HET^ CD4^+^ T cells.

### HDAC1^cKO^-HDAC2^HET^ mice induce CD4^+^ CTL features in Th cells upon murine cytomegalovirus infection.

**Figure 6:**
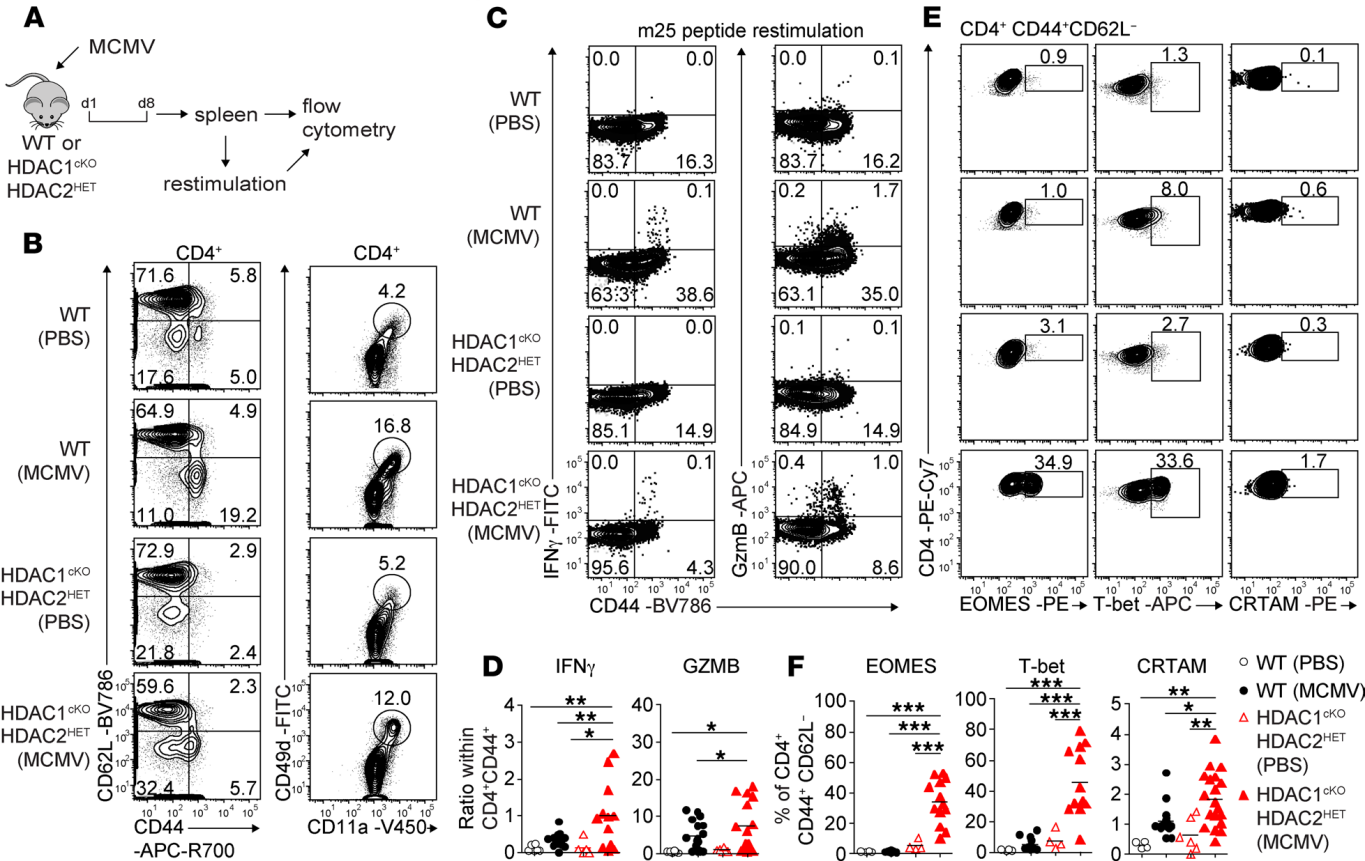
Enhanced induction of CD4^+^ CTLs in HDAC1^cKO^-HDAC2^HET^ mice upon MCMV infection. (**A**) Experimental strategy: WT and HDAC1^cKO^-HDAC2^HET^ mice were intraperitoneally injected with 5 × 10^5^ PFU/mouse Δm157-MCMV. Eight days after infection the spleen was isolated, and single-cell suspensions were analyzed by flow cytometry or, prior, restimulated with viral m25 peptide for 4 hours. (**B**) Flow cytometry analysis of splenocytes isolated from MCMV-infected (or PBS-injected controls) WT and HDAC1^cKO^-HDAC2^HET^ mice showing CD44, CD62L, CD11a, and CD49d expression on TCRβ^+^CD4^+^ cells. (**C**) Contour plots depict IFN-γ and granzyme B expression in WT and HDAC1^cKO^-HDAC2^HET^ TCRβ^+^CD4^+^ T cells (isolated from MCMV-infected or control mice) restimulated with m25 viral peptide. (**D**) Summary of experiments described in **C**. Diagrams depict the ratio of the percentages of either IFN-γ^+^CD4^+^CD44^hi^ or granzyme B–positive CD4^+^CD44^hi^TCRβ^+^ cells to the percentages of all CD44^hi^ cells within the TCRβ^+^CD4^+^ T cell population isolated from MCMV-infected and control WT and HDAC1^cKO^-HDAC2^HET^ mice. (**E**) Contour plots show EOMES, T-bet, and CRTAM expression on TCRβ^+^CD4^+^CD44^+^CD62L^–^ splenocytes isolated from WT and HDAC1^cKO^-HDAC2^HET^ (MCMV-infected or control) mice. (**F**) Summary of experiments described in **C**. Diagrams depict the percentages of TCRβ^+^CD4^+^CD44^+^CD62L^–^ splenocytes expressing EOMES, T-bet, and CRTAM. (**B**, **C**, and **E**) Numbers indicate the percentages of cells in the respective quadrants or gates. (**D** and **F**) Each symbol indicates 1 mouse. Horizontal bars indicate the mean. *P < 0.05, **P < 0.01, and ***P < 0.001 (1-way ANOVA analysis followed by Tukey’s multiple-comparisons test). Data are representative (**B**, **C**, and **E**) or show a summary (**D** and **F**) of at least 16 (**B**, **C**, and **E**) mice that were analyzed in at least 4 (**B**, **E**, and **F**) or 3 (**C** and **D**) independent experiments.

Next we determined whether HDAC1/HDAC2 also control CD4^+^ CTL generation in response to viral infection in vivo. We used a murine cytomegalovirus (MCMV) model (Figure 6A) because MCMV induces, in addition to a strong CD8^+^ T cell response, the generation of CD4^+^ T cells with CTL activity (33–35). Infected WT mice showed an increase in the percentage and number of splenic CD8^+^ T cells on day 8 (Supplemental Figure 5, A and B). Further, CD44^hi^CD62L^–^ cells and CD11a^+^CD49d^+^ effector cells within the CD4^+^ T cell subset were increased, indicative of a CD4^+^ T cell response (35) (Figure 6B and Supplemental Figure 5, B and C). Restimulation of splenocytes with the MHC class II immune-dominant MCMV m25 peptide (33) induced IFN-γ and granzyme B expression in CD4^+^CD44^hi^ cells, while CD4^+^ T cells from noninfected mice did not produce IFN-γ (Figure 6, C and D). Moreover, the fraction of CRTAM-expressing CD4^+^CD44^hi^ T cells was enhanced, and we observed a tendency toward increased EOMES^+^ and T-bet^+^ CD4^+^CD44^hi^ T cells in comparison with CD4^+^CD44^hi^ T cells of PBS-injected control mice (Figure 6, E and F), indicating induction of CD4^+^ CTL subsets upon MCMV infection. In MCMV-infected HDAC1^cKO^-HDAC2^HET^ mice the fraction of CD11a^+^CD49d^+^ cells within CD4^+^ T cells was also expanded in comparison with noninfected HDAC1^cKO^-HDAC2^HET^ mice, while the percentages of neither CD4^+^CD44^hi^ nor CD8^+^ T cells were increased (Figure 6B and Supplemental Figure 5B). Although the percentages of IFN-γ–producing or granzyme B–expressing CD4^+^ T cells were lower in HDAC1^cKO^-HDAC2^HET^ mice compared with WT mice (Figure 6C), the fraction of IFN-γ– and also granzyme B–producing cells within the CD4^+^CD44^hi^ population was higher in infected HDAC1^cKO^-HDAC2^HET^ mice than in infected WT mice (Figure 6, C and D). This suggests enhanced activation of CD4^+^ T cells but impaired effector CD4^+^ T cell expansion in HDAC1^cKO^-HDAC2^HET^ mice. Nevertheless, there was a strong increase in the percentage of cells that expressed CRTAM, EOMES, and T-bet within the CD4^+^CD44^hi^ T cells in HDAC1^cKO^-HDAC2^HET^ mice (Figure 6, E and F), suggesting enhanced induction of CD4^+^ CTL features in HDAC1^cKO^-HDAC2^HET^ Th cells upon infection. Together, these data indicate that HDAC1/HDAC2 control the extent of CD4^+^ CTL induction in response to MCMV infection in vivo.

### Human CD4^+^ T cells treated with the class I HDACi MS-275 upregulate CTL features.

**Figure 7:**
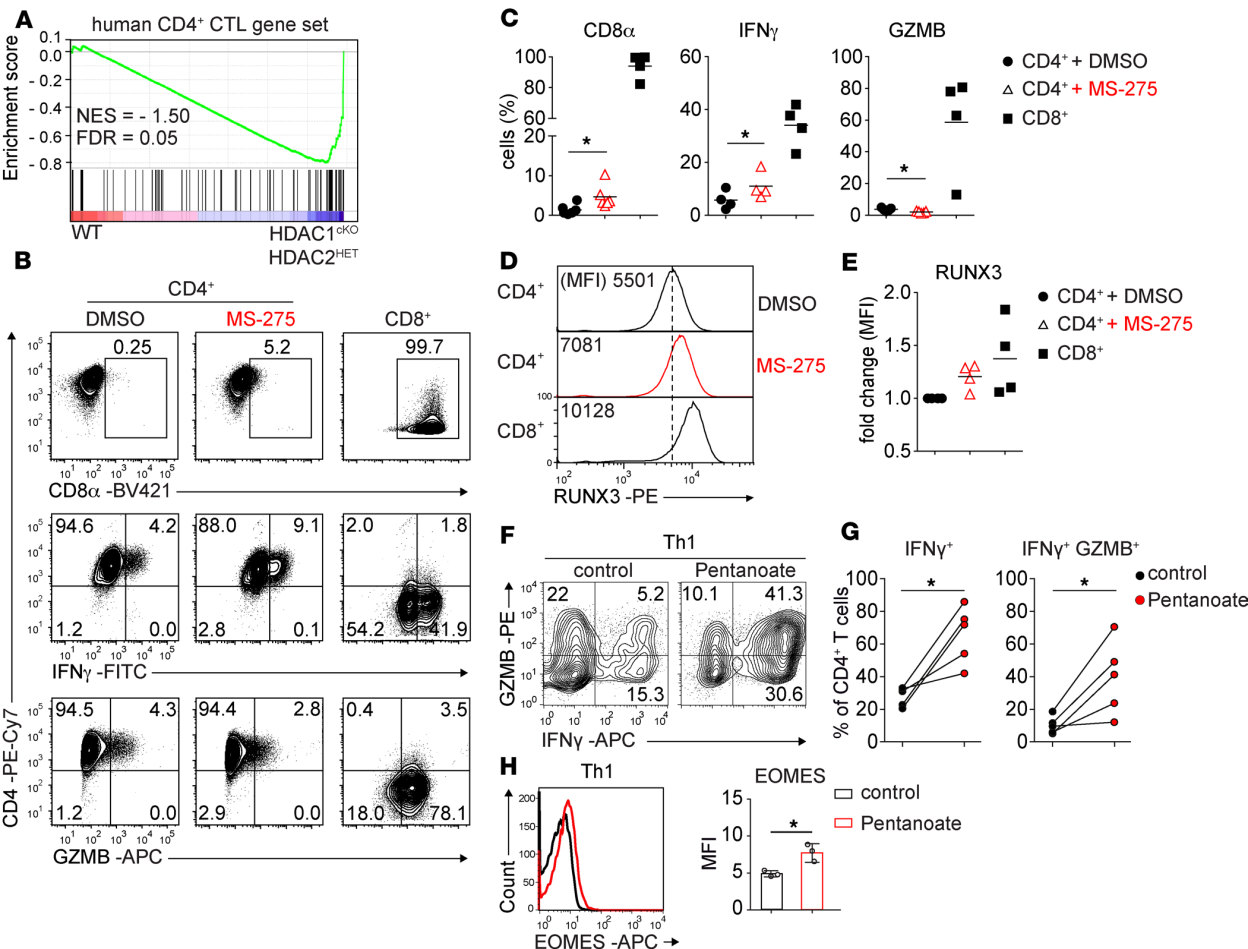
Human CD4^+^ T cells upregulate Th cytotoxic genes upon HDACi treatment. (**A**) GSEA plots of human CD4^+^ CTL-specific gene sets (containing 517 genes) in activated HDAC1^cKO^-HDAC2^HET^ CD4^+^ T cells relative to activated WT CD4^+^ T cells. The barcode indicates the location of the members of the gene set in the ranked list of all genes. (**B**) Flow cytometry analysis showing CD4, CD8α, IFN-γ, and granzyme B expression in naive human CD4^+^ and CD8^+^ T cells (right panel) activated with CD3/CD28 Dynabeads for 5 days, for the last 24 hours in the presence of DMSO (left panel) and MS-275 (middle panel), respectively. (**C**) Summary of experiments described in **A**. Diagrams depict the percentages of activated CD4^+^ and CD8^+^ T cells, respectively, expressing CD8α, IFN-γ, and granzyme B. (**D**) Histogram panel depicts RUNX3 expression in activated CD4^+^ T cells (as described in **A**) treated with DMSO (upper panel) or MS-275 (middle panel) for 24 hours and untreated CD8^+^ T cells as staining control (lower panel). (**E**) Summary of the experiment described in **C**. Diagram depicts the FC of MFI of RUNX3 expression in activated CD4^+^ and CD8^+^ T cells, respectively. (**F** and **H**) Flow cytometry analysis showing IFN-γ, granzyme B, and EOMES expression on human Th1 cells cultured in the absence (control) or presence of pentanoate. Diagrams depict the summary of (**G**) the percentages of IFN-γ^+^ and IFN-γ^+^GZMB^+^ human Th1 cells, respectively, as well as (**H**) MFI of EOMES expression in human Th1 cells. Data are representative (**B**, **D**, **F**, and **H**) or show a summary (**C**, **E**, **F**, **G**) of 5 (**B** and **C** for CD8α), 4 (**B**–**D** for IFN-γ, GZMB, RUNX3), or 3 (**H**) human donors who were analyzed in 2 (**F–H**), 5 (**B** and **C** for CD8α), or 4 (**B–E** for IFN-γ, GZMB, RUNX3) independent experiments. Numbers indicate the percentages of cells in the respective quadrants or gates (**B** and **F**) or MFI (**D**). (**D**) The dotted vertical lines indicate the peak of the WT histogram (for MFI). (**C**, **E**, **G**, and **H**) Each symbol indicates 1 human donor. Horizontal bars indicate the mean. *P < 0.05 (paired 2-tailed Student’s *t* test).

A recent single-cell RNA-Seq analysis of human CD45RA^+^IL-7R^–^CD4^+^ effector memory T cells (IL-7R^–^CD4^+^ TemRA) identified a set of 517 genes upregulated in IL-7R^–^CD4^+^ TemRA cells in comparison with CD4^+^ central memory T (Tcm) cells. Among these 517 genes an overrepresentation of a cytotoxicity gene signature was observed, and hence IL-7R^–^CD4^+^ TemRA cells have been defined as CD4-CTL effector cells (compared with Tcm) (36). By performing GSEA we observed that murine homologs of this human CD4-CTL effector gene list were enriched in the list of differentially expressed genes of HDAC1^cKO^-HDAC2^HET^ CD4^+^ T cells (Figure 7A), substantiating our finding that a cytotoxic gene signature was induced in HDAC1^cKO^-HDAC2^HET^ CD4^+^ T cells. To test whether HDAC inhibition leads to an upregulation of CD4^+^ CTL features in human T cells also, we activated freshly isolated naive human CD4^+^ T cells for 5 days with anti-CD3/anti-CD28 and added the HDACi MS-275 (or DMSO as a carrier control) during the last 24 hours of the culture period. We observed an induction of CD8α expression and an increase in the percentage of IFN-γ–producing cells, although granzyme B was not induced in MS-275–treated human CD4^+^ T cells (Figure 7, B and C). Moreover, RUNX3 expression was upregulated in the presence of MS-275 (Figure 7, D and E). These data indicate a potential role of class I HDACs in certain aspects of human CD4^+^ CTLs’ differentiation.

### Short-chain fatty acid treatment induces cytotoxic features in murine and human CD4^+^ T cells.

Short-chain fatty acids (SCFAs), which in part also function as HDACis (37), are commensal-produced metabolites that have an impact on differentiating T cells (38, 39). To test whether these physiologically generated HDACis have the potential to induce CTL features in CD4^+^ T cells, we activated murine WT CD4^+^ T cells with anti-CD3/anti-CD28 for 3 days in the presence of the SCFAs acetate, propionate, butyrate, or pentanoate. We observed a strong induction of IFN-γ, granzyme B, and T-bet as well as a weak induction of CD8α and EOMES by propionate, butyrate, and pentanoate (Supplemental Figure 6, A–C). Moreover, human CD4^+^ T cells activated in the presence of pentanoate showed a strong upregulation of IFN-γ, granzyme B, and EOMES (Figure 7, F–H). Together, these data indicate that SCFAs promote the generation of CD4^+^ T cells with CTL features.

## Discussion

In this study we demonstrate that HDAC1-HDAC2 expression levels and thus their combined activity are a key determinant for CD4^+^ CTL differentiation. We previously reported that HDAC1 and HDAC2 maintain the integrity and regulate the survival of the CD4 lineage and showed plasticity of activated HDAC1-2^cDKO^ CD4^+^ T cells toward the CD8 lineage ex vivo (14). In the present study we revealed, using mice with graded *Hdac1* and/or *Hdac2* gene dosages, that either 1 *Hdac1* or 1 *Hdac2* allele is sufficient to support the survival of activated CD4^+^ T cells, similar to observations made in immature double-negative thymocytes (40). This opened up an experimental system to study and dissect the impact of HDAC1/HDAC2 for CD4^+^ CTL differentiation. Moreover, our observation of a gene dosage–dependent effect of *Hdac1* and *Hdac2* in restricting the induction of cytotoxic features in vitro showed that HDAC1 is more potent than HDAC2 in repressing the extent of CD4^+^ CTL features in Th cells. This suggests different roles for HDAC1 and HDAC2 in CD4^+^ T cells. Of note, a similar differential requirement for either HDAC1 or HDAC2, despite being expressed in both lineages, has also been reported for epidermis (41) and neuronal cells (42), respectively.

Our study provides insight into signaling and gene regulatory networks driving CD4^+^ CTL differentiation (Supplemental Figure 7). The induction of CD4^+^ CTL features in HDAC1^cKO^-HDAC2^HET^ CD4^+^ T cells was accompanied by a strong upregulation of CRTAM, which directs CD4^+^ CTL differentiation by inducing the expression of IFN-γ and CTL-related genes as well as by promoting cytotoxic activity (9). This suggests that a key step in CD4^+^ CTL induction is an HDAC1/HDAC2-controlled regulation of *Crtam* expression in CD4^+^ T cells. Moreover, the reprogramming of CD4^+^ T cells into intestinal intraepithelial CD4^+^ CTLs is accompanied by a downregulation of the CD4 lineage commitment factor ThPOK and a corresponding upregulation of RUNX3, a key factor for the development of CD8 lineage T cells (21, 22). A similar crossregulation of RUNX3 and ThPOK was also observed during the acquisition of cytotoxic function by human Th1 lymphocytes in the context of human cytomegalovirus infection (2). We previously showed an upregulation of RUNX3 in HDAC1-2^cDKO^ CD4^+^ T cells and that HDAC1/2-mediated repression of RUNX/CBFβ is required to maintain CD4 lineage integrity (14). A crucial role for ThPOK was revealed by the observation that combined deletion of ThPOK and LRF in postthymic CD4^+^ T cells results in transdifferentiation of mature CD4^+^ T cells into CD8 lineage T cells (19). In line with these studies, we observed similar dynamic changes in RUNX3 and ThPOK expression levels in activated HDAC1^cKO^-HDAC2^HET^ CD4^+^ T cells that acquire cytolytic activity in vitro. Because enforced expression of ThPOK in HDAC1^cKO^-HDAC2^HET^ CD4^+^ T cells blocked CRTAM upregulation, it is tempting to speculate that ThPOK downregulation is a prerequisite for *Crtam* induction. However, ThPOK overexpression only partially blocked CRTAM induction and upregulation of CTL genes and was not sufficient to downregulate T-bet and EOMES expression in activated HDAC1^cKO^-HDAC2^HET^ CD4^+^ T cells. Our data imply that additional pathways, independent of ThPOK-mediated repression, are required for CD4^+^ CTL differentiation. An IFN-γ–JAK1/2–STAT1 signaling axis might be such a candidate pathway because IFN-γ–blocking experiments and JAK inhibitor treatment revealed that IFN-γ and downstream signaling pathways are key to but not sufficient to induce Th cytotoxic features in HDAC1^cKO^-HDAC2^HET^ CD4^+^ T cells. Our observation that IFN-γ–JAK1/2–STAT1 signaling is crucial for CTL induction is also in agreement with a study showing that the upregulation of IFN-γ and T-bet expression is important for intestinal intraepithelial lymphocytes’ differentiation, which contain populations of CD4^+^ CTLs (43). Whether other JAK/STAT members control CD4^+^ CTL induction remains to be determined. The JAK3 inhibitor tofacitinib partially blocked the induction of granzyme B and RUNX3; however, tofacitinib inhibits JAK1 also (44). Thus we cannot rule out that tofacitinib’s effect is due to JAK1 inhibition. Further, the induction of Th cytotoxic genes in HDAC1^cKO^-HDAC2^HET^ CD4^+^ T cells was controlled by T cell–intrinsic mechanisms and not due to the secretion of soluble factors or the expression of surface molecules that affect neighboring cells. Thus, we propose that HDAC1 and HDAC2 regulate the extent of CD4^+^ CTLs’ induction via controlling IFN-γ–JAK1/2–STAT1 signaling as well as RUNX3/ThPOK transcription factor–dependent networks that also govern the induction of CRTAM. We detected *Runx3* upregulation and *Zbtb7b* downregulation at the mRNA level, suggesting that HDAC1/HDAC2 regulate these genes at the transcriptional level. Nevertheless, we cannot exclude the possibility that HDAC1/HDAC2 regulate these transcription factors also at a posttranslational level, since both ThPOK (45) and RUNX3 (46) are targets of reversible lysine acetylation. Similarly, STAT1 is reversibly lysine acetylated and interacts with HDAC1 (47, 48), suggesting a potential impact of HDAC1/HDAC2 on STAT1 protein. Future studies addressing the HDAC1/HDAC2-dependent acetyl-proteome are required to reveal the contribution of posttranslational modifications of RUNX3, ThPOK, and STAT1 in the induction of CD4^+^ CTLs.

In summary, our data provide mechanistic insight into molecular mechanisms of CD4^+^ CTL differentiation. Further, our results suggest that SCFAs might be physiological inducers of CD4^+^ CTL induction. Increasing numbers of studies highlight the importance of CD4^+^ CTLs in chronic viral inflammation and cancer immunity (1, 8). Moreover, CD4^+^ chimeric antigen receptor T cells display cytolytic activity (49, 50), implicating cytotoxic functions of CD4^+^ T cells in cancer immunotherapy. Thus, our results also imply that a transient application of class I HDACis might be a promising strategy for the induction of a CD4^+^ CTL response in a therapeutic setting. Additional studies are warranted to explore this further.

## Methods

### Mice.

*Hdac1*^fl/fl^*Hdac2*^fl/fl^ (HDAC1-2^cKO^) *Cd4*-Cre mice (Mouse Genome Informatics [MGI] 4440556 for *Hdac1*; MGI 4440560 for *Hdac2*) (14) and *Stat1*^fl/fl^ (MGI 5319173) (51) and *Stat1*^fl/fl^ *Lck*-Cre mice (52) were described previously. *Cd4*-Cre mice (MGI 2386448) were described previously (53). Crossing of HDAC1-2^cKO^ to either *Hdac1*^fl/fl^ *Cd4*-Cre or *Hdac2*^fl/fl^ *Cd4*-Cre generated HDAC1^cKO^-HDAC2^HET^ and HDAC1^HET^-HDAC2^cKO^ mice, respectively. CD45.1^+^ C57BL/6 congenic mice were obtained from the European Mouse Mutant Archive (EM 01998). Mice of both sexes were analyzed between 6 and 12 weeks of age, unless otherwise stated. Littermate controls were used for all experiments and for flow cytometry analysis within 1 experiment.

### Purification of naive CD4^+^ T cells.

Cells from spleen, axillary, brachial, and inguinal lymph nodes were pooled and incubated with a cocktail of biotinylated antibodies (anti–mouse CD11b [RRID: AB_312787; clone: MEL1/70], anti–mouse CD11c [AB_313773; N418], anti–mouse B220 [AB_312988; RA3-6B2], anti-mouse Gr1 [AB_313369; Ly-6g], anti–mouse NK1.1 [AB_313391; PK136], anti–mouse Ter-119 [AB_313705; Ter-119], and anti–mouse CD8α [AB_312743; 53-6.7] or CD4 [AB_312710; RM4-5] in PBS/2% FBS; all from BioLegend). CD4^+^ T cells were enriched by negative depletion using magnetic streptavidin beads (MagniSort SAV Negative Selection beads, Thermo Fisher Scientific) according to the manufacturer’s instructions. Cells were further sorted for naive CD4^+^ T cells (CD25^–^CD44^lo^CD62L^+^) on a BD FACSAriaII (BD Biosciences) or on an SH800 (SONY).

### T cell activation and cell proliferation analysis.

For Th0 conditions, FACS-sorted naive CD4^+^ T cells were stimulated (day 0) with plate-bound anti-CD3ε (1 μg/mL; AB_394590; BD Biosciences) and anti-CD28 (3 μg/mL; AB_394763; BD Biosciences) on 48-well plates (0.2 × 10^6^ cells/well) in 1 mL T cell medium/well (RPMI1640 supplemented with 10% FCS [MilliporeSigma/Biowest], antibiotics, 50 mM β-ME) supplemented with 20 U/mL recombinant human IL-2 (rhIL-2, PeproTech) for 3 days, unless otherwise stated. Th1 cells were generated from sorted naive CD4^+^ T cells activated with anti-CD3ε/anti-CD28 in the presence of 20 U/mL rhIL-2 (PeproTech), 5 ng/mL IL-12 (R&D Systems), and 3 μg/mL anti–IL-4 (BioXcell) for 3 days. For assessment of cell proliferation, naive CD4^+^ T cells were labeled using Cell Proliferation Dye eFluor 450 (Thermo Fisher Scientific) according to the manufacturer’s protocol before activation. Cells were harvested and analyzed on day 3 unless otherwise indicated. For cytokine detection, activated cells were restimulated for 4 hours with phorbol 12-myristate 13-acetate (PMA, 25 ng/mL) and ionomycin (Iono, 750 ng/mL), both from MilliporeSigma, in the presence of GolgiStop (BD Biosciences). CD8^+^ T cells were activated and cultured with anti-CD3ε/anti-CD28 as described above in the presence of 40 U/mL rhIL-2.

### Extracellular and intracellular stainings.

Two million cells were incubated with Fc Block (1:250; BD Biosciences) followed by surface staining. Dead cells were excluded using Fixable Viability Dye eFluor 506 (Thermo Fisher Scientific) according to the manufacturer’s protocol. For intracellular cytokine stainings, cells were fixed with Cytofix Fixation Buffer (BD Biosciences), permeabilized with Perm/Wash Buffer (BD Biosciences), and stained according to the manufacturer’s protocol. For intracellular transcription factor stainings, cells were fixed and permeabilized using the Foxp3 Staining Buffer Set (Thermo Fisher Scientific) according to the manufacturer’s protocol and stained with the appropriate antibodies. Cells were measured with a BD LSRFortessa or BD LSRII cytometer and analyzed using FlowJo 10.2 software. The following antibodies were used: CD90.2 (AB_1272223; 30-H12), B220 (AB_465052; RA3-6B2), TCRβ (AB_1272173; H57-597), eomesodermin (AB_1603274; Dan11mag), CD8α (AB_469335; 53-6.7), T-bet (AB_2744712; 4B10), IFN-γ (AB_465412; XMG1.2), and granzyme B (AB_2536539; GB11), all from Thermo Fisher Scientific; CD25 (AB_395101; PC61), CD34 (AB_1645242; RAM34), CD69 (AB_1727511; H1.2F3), CD8α (AB_394081; 53-6.7), CD8β (AB_393887; H35-17.2), CD49d (AB_394669; R1-2), IL-2 (AB_395386; JES6-5H4), RUNX3 (AB_2738969; R3-5G4), and ThPOK (AB_2739268; Zbtb7B, T43-94), all from BD Biosciences; and CD45.1 (AB_2564295; A20), CD24 (AB_312840; M1/69), CD62L (AB_2563058, AB_2629685; MEL-14), and CRTAM (AB_2085907; CD355, 11-5, all from BioLegend. For detection of early and late apoptotic cells, a 7-AAD/annexin V staining kit (Thermo Fisher Scientific) was used according to the manufacturer’s protocol.

### Intracellular HDAC1 and HDAC2 staining.

Splenocytes were isolated, incubated with Fc Block (BD Biosciences), and stained with TCRβ, CD8α, and CD4 antibodies. Cells were fixed and permeabilized using Foxp3 Staining Buffer Set, blocked in 5% normal goat serum, and incubated with rabbit anti–mouse HDAC1 (AB_10918369; ABE260) and mouse anti–mouse HDAC2 (AB_310022; 3F3) antibodies in permeabilization buffer (Thermo Fisher Scientific) for 1 hour. Subsequently, cells were washed with permeabilization buffer and further incubated with Alexa Fluor 488–conjugated goat anti–rabbit IgG1 (1 hour) (AB_142134; Invitrogen, Thermo Fisher Scientific) and biotinylated anti–mouse IgG1 antibodies (1 hour) (AB_394861; BD Biosciences), followed by a streptavidin secondary staining (AB_2571915; BioLegend).

### Intracellular detection of STAT1 phosphorylation.

Sorted naive CD4^+^ T cells were activated as described above for 24, 48, and 72 hours. Subsequently, cells were incubated with Cytofix fixation buffer (BD Biosciences) for 20 minutes at 37°C followed by a 20-minute incubation with cooled (–20°C) BD Phosflow Perm Buffer III (BD Biosciences). Afterward, cells were washed with PBS/2% FCS and stained with anti–p-STAT1 (PY701) (AB_399855; clone 4a) or anti–p-STAT5 (pY694, AB_399882; clone 47) (both from BD Biosciences), followed by flow cytometry analysis.

### Detection of LAMP protein (CD107a) by flow cytometry.

Naive CD4^+^ T cells were cultured under Th0 conditions as described above. After 3 days, cells were incubated with Fc Block (BD Biosciences) and restimulated with PMA/Iono. Fluorochrome-conjugated antibodies against CD107a (AB_395057; 1D4B, BD Biosciences) were added at the beginning of restimulation along with GolgiStop (1:250) and GolgiPlug (1:250, BD Biosciences). After the stimulation period cells were washed with PBS/2% FCS, stained with anti-CD4 and -CD8 antibodies (as described above), and analyzed by flow cytometry.

### In vitro cytotoxicity assay.

For redirected cytotoxicity assays, naive CD4^+^ T cells were activated under Th0 conditions as described above for 3 days in the presence of 20 U/mL rhIL-2 to generate activated effector CD4^+^ T cells. Fc receptor–positive P815 target cells (ATCC, TIB-64) were mixed with 2 × 10^5^ activated effector CD4^+^ T cells at 1:1, 1:3, and 1:10 ratios and incubated at 37°C for 4 hours in 200 μL T cell medium in the presence of soluble anti-CD3ε antibody (10 μg/mL) in a 96-well, V-shaped plate. After the incubation period, the cell mixture was stained with annexin V, and target cells (CD34^+^CD90.2^–^) were quantified for the apoptotic marker by flow cytometry. As a negative control, effector and target cells were co-incubated without anti-CD3ε.

### JAK1/3 inhibitor experiments.

Sorted naive CD4^+^ T cells were labeled with proliferation dye and activated under Th0 conditions as described above in the presence or absence of either 10 nM ruxolitinib or 10 nM tofacitinib (both Selleckchem). At day 3, extracellular and intracellular stainings were performed, and cells were analyzed by flow cytometry.

### Exogenous addition of IFN-γ and in vitro IFN-γ blocking.

Sorted naive CD4^+^ or CD8^+^ T cells were activated under Th0 conditions and cultured for 3 days in the presence of either 300 ng/mL exogenous IFN-γ (PeproTech) or 20 μg/mL neutralizing IFN-γ antibody (AB_1107692; BioXcell). On day 3, cells were stained and analyzed using flow cytometry.

### Coculture experiments.

One hundred thousand naive WT CD4^+^ T cells isolated from CD45.1^+^ C57BL/6 congenic mice were cocultured with 1 × 10^5^ naive CD45.2^+^ CD4^+^ T cells isolated from either WT or HDAC1^cKO^-HDAC2^HET^ mice in the presence of 20 U/mL rhIL-2 for 3 days in a 48-well plate and subsequently analyzed using flow cytometry.

### HDACi experiments.

CD4^+^ T cells were isolated from spleens and lymph nodes of *Stat1*^fl/fl^ *Lck*-Cre and *Stat1*^fl/fl^ mice and activated under Th0 conditions. MS-275 (Selleckchem) (2 μM final concentration) or DMSO only (as carrier control) was added 48 hours later, and cells were cultured for an additional 24 hours. Afterward, cells were stained and analyzed using flow cytometry. For SCFA treatment, CD4^+^ T cells were isolated from spleens and lymph nodes of WT mice and activated under Th0 conditions in the presence/absence of SCFAs for 72 hours. Acetate (Sigma-Aldrich), propionate (Sigma-Aldrich), and pentanoate (Merck) were added to a final concentration of 2 mM. Butyrate (Sigma-Aldrich) was added to a final concentration of 0.75 mM. Afterward, cells were stained and analyzed using flow cytometry.

### Retroviral expression of ThPOK.

High-titer viral preparations were generated as previously described (14). Briefly, Phoenix-E packaging cells (provided by Garry Nolan, Stanford University, Stanford, California, USA) cultured in 10-cm dishes in DMEM (supplemented with 10% FCS and antibiotics) were transiently transfected with 20 μg ThPOK retroviral vector (MSCV-FH-ThPOK-IRES-EGFP; provided by Ichiro Taniuchi, RIKEN Center for Integrative Medical Sciences, Yokohama, Japan) and 20 μg “empty” MSCV-IRES-EGFP CTRL using standard calcium phosphate precipitation. Twenty-four hours after transfection, the medium was changed into T cell medium. One day later, viral supernatants were collected, filtered through a 45-μm filter, and used to infect CD4^+^ T cells. Then, 0.2 × 10^6^ naive CD4^+^ T cells/mL were sorted and activated under Th0 conditions in the presence of 20 U/mL rhIL-2 for 3 days. On day 3 of CD4^+^ T cell culture, the culture medium was removed, and 1 mL virus-containing supernatant containing 10 mg polybrene (H9268, MilliporeSigma) was added per well (48-well plate). Spin infection was performed at 6000 *g* for 2 hours at 32°C; cells were then placed into the same 1 mL of T cell medium containing rhIL-2 and cultured for 1 additional day. On culture day 4, cells were analyzed by flow cytometry.

### MCMV infection.

The Δm157-MCMV strain (54) was grown in mouse embryo fibroblasts, purified through a sucrose cushion, and passed through a 0.45-μm pore size filter. Mice were infected with 5 × 10^5^ PFU/mouse Δm157-MCMV intraperitoneally or injected with PBS intraperitoneally, as controls. On day 8 after infection, the spleen was isolated. For lymphocyte isolation from liver tissue, a Percoll gradient centrifugation step was performed. Two million cells were used for extracellular and intracellular staining followed by direct flow cytometry analysis. For restimulation, the following MHC class II–restricted MCMV peptide epitope m25 (NHLYETPISATAMVI) (33) was used. Four million cells/well were incubated with 3 μg/mL of the respective peptide in a 48-well plate for 6 hours, for the last 4 hours in the presence of GolgiStop, followed by flow cytometry analysis.

### Isolation, culture, and analysis of human CD4^+^ and CD8^+^ T cells.

Pan-T cells were isolated from 30 mL whole blood using human MACSxpress Whole Blood Pan T Cell Isolation Kit (Miltenyi Biotec). Cells were extracellularly stained using anti-CD4 (AB_314079; RPA-T4), -CD8α (AB_10898322; RPA-T8), -CD45RA (AB_893358; HI100), -CD45RO (AB_2566542; UCHL1), -CCR7 (AB_10913812; G043H7), and -CD25 (AB_2561860; M-A251), all from BioLegend, followed by FACS sorting for naive (CCR7^+^CD45RA^+^CD45RO^–^) CD4^+^ T cells and CD8^+^ T cells, respectively, on an SH800. FACS-sorted naive CD4^+^/CD8^+^ T cells were stimulated with Dynabeads Human T-activator CD3/CD28 (Gibco, Thermo Fisher Scientific) at a 1:1 ratio on 96-well plates (75,000 cells/well) in 200 μL AIM V medium/well (Gibco, Thermo Fisher Scientific) for 5 days, in the last 24 hours in the presence of DMSO and 2 μM MS-275, respectively. For cytokine detection, activated cells were restimulated for 4 hours with PMA (25 ng/mL) and Iono (750 ng/mL), both from Sigma-Aldrich, in the presence of GolgiStop (BD Biosciences). For the treatment with SCFAs, FACS-sorted naive CD4^+^ T cells were stimulated with T Cell Activation MACSiBeads (Miltenyi Biotec) in presence of 20 ng/mL rhIL-12 for 5 days. Twenty-four hours after stimulation with beads, pentanoate was added to a final concentration of 3 mM. For cytokine detection, activated cells were restimulated for 4 hours with PMA and Iono in the presence of Brefeldin A (5 μg/mL, BioLegend). Subsequently, approximately 0.5 × 10^6^ cells were surface stained using CD4 and CD8α; dead cells were excluded using Fixable Viability Dye eFluor 506 (Thermo Fisher Scientific) according to the manufacturer’s protocol. For intracellular cytokine stainings, cells were fixed with Cytofix Fixation Buffer (BD Biosciences), permeabilized with Perm/Wash Buffer (BD Biosciences), and stained according to the manufacturer’s protocol. For intracellular transcription factor stainings, cells were fixed and permeabilized using the Foxp3 Staining Buffer Set (Thermo Fisher Scientific) according to the manufacturer’s protocol. Cells were stained using the following antibodies: IFN-γ (AB_315230, AB_315236; 4S.B3) from BioLegend, RUNX3 (AB_2738969; R3-5G4) and granzyme B (AB_11154033; GB11) from BD Biosciences, and EOMES (AB_2574229; WD1928) from Thermo Fisher Scientific. Cells were measured with a BD LSRFortessa or BD LSRII cytometer and analyzed using FlowJo 10.2 software.

### RNA-Seq and sample preparation.

Sorted naive CD4^+^ T cells were cultured under Th0 conditions in the presence of rhIL-2 as described above. After 3 days, cells were harvested and stained with 7-AAD for 5 minutes, and alive cells were purified by FACS sorting. Total RNA was prepared from approximately 2 × 10^6^ cells and isolated using RNeasy Mini Kit (QIAGEN Inc.), including an on-column DNA digestion step (RNAse-Free DNAse Set, QIAGEN Inc.). RNA amount was measured using Qubit 2.0 Fluorometric Quantitation (Life Technologies), and RNA integrity number was determined using Experion Automated Electrophoresis System (Bio-Rad). RNA-Seq libraries were generated using a Sciclone NGS Workstation (PerkinElmer) and a Zepyhr NGS Workstation (PerkinElmer) with the TruSeq Stranded mRNA LT sample preparation kit (Illumina). Library amount and quality were determined using Qubit 2.0 and Automated Electrophoresis System (Bio-Rad). The libraries were sequenced by the Biomedical Sequencing Facility at CeMM using the Illumina HiSeq 3000 platform and the 50-bp single-read configuration. The RNA-Seq data have been deposited in the National Center for Biotechnology Information’s Gene Expression Omnibus database under number GSE134368.

### Bioinformatic analysis.

Raw sequencing data were processed with Illumina2 bam-tools 1.17 to generate sample-specific, unaligned BAM files. Sequence reads were mapped onto the mouse genome assembly build mm10 (a flavor of GRCm38) using TopHat 2.0.13 (55). Gene expression values (reads per kilobase exon per million mapped reads) were calculated with Cufflinks 2.2.1 (56). The volcano plot was generated using GraphPad Prism. The downstream pathway analysis was performed using the GSEA tools provided by the Broad Institute (57, 58) or Ingenuity Pathway Analysis (QIAGEN Inc.) (59).

### “Th1-selective” and “CTL-selective” gene sets.

Gene expression microarray data (Affymetrix Mouse Exon 1.0 ST) for WT Th1 cells and WT CD8^+^ T cells (cultures under Th1 conditions) were taken from Vacchio et al. (19) and normalized using the robust multiarray average method. Transcriptome data were compared and 168 genes preferentially expressed in WT Th1 cells (“Th1 gene set”) and 477 genes preferentially expressed in CD8^+^ T cells (“CD8-lineage gene set”) were defined using the R/Bioconductor package limma (FC ≥ 2; FDR ≤ 0.05).

### Data availability.

RNA-Seq data have been deposited in the Gene Expression Omnibus database (GSE134368). The published human CD4^+^ CTL gene sets (Figure 7A) were taken from Patil et al. (36). Microarray data from Th1 and activated CD8^+^ T cells (Figure 2D) were taken from Vacchio et al. (19).

### Statistics.

No statistical methods were used to predetermine the sample size. All statistical analyses were performed using Prism Software (GraphPad). Dependent on the experimental setup, *P* values were calculated with an unpaired 2-tailed Student’s *t* test (a normal distribution of data points was assumed; variances were assessed, and if necessary an unpaired *t* test with Welch’s correction was applied) or with a 1-way ANOVA analysis followed by Tukey’s multiple-comparisons test. The *P* values were defined as *P < 0.05; **P < 0.01; ***P < 0.001. Differences that did not reach a statistically significant level (i.e., *P* ≥ 0.05) were either indicated as “n.s.” for 2-group comparisons or not indicated for multiple-group comparisons. No data were excluded.

### Study approval.

The usage of human blood samples was approved by the ethics committees of the Medical University of Vienna (EK 1344_2018) and conducted in accordance with the Declaration of Helsinki. Healthy blood donors gave written informed consent. Animal husbandry and experimentation were performed under the national laws and approved by the Austrian Federal Ministry for Science and Research, Vienna, Austria, and ethics committees of the Medical University of Vienna (BMWFW-66.009/0105-WF/II/3b/2014 and GZ:BMBFW-66.009/0039-WF/II/3b/2019) and according to the guidelines of the Federation of European Laboratory Animal Science Associations, which match that of Animal Research: Reporting of In Vivo Experiments. MCMV infection experiments were performed at the University of Veterinary Medicine Vienna and were approved by the institutional ethics and animal welfare committee and the national authority according to §26ff. of the Animal Experiments Act, Tierversuchsgesetz 2012 (BMWFW 68.205/0032-WF/II/3b/2014).

## Author contributions

TP and WE designed the research; TP performed most of the experiments and analyzed the data; ML, PH, T Bulat, LA, LG, T Boenke, VS, RR, LS, DW, and RT performed some of the experiments and analyzed data; BS provided reagents and analyzed data; AV, NB, and BB designed some experiments and analyzed data; RT, GS, TD, and CS provided reagents and mice; TF, LLE, AL, and SS provided data sets; T Boenke prepared all RNA-Seq libraries; and CB supervised the RNA-Seq experiments and data analysis. TP and WE wrote the manuscript with contributions from all coauthors.

## Supplementary Material

Supplemental data

## Version 1 02/27/2020

Electronic publication
