# Supplementary material for: Histone deacetylases 1 and 2 restrain CD4+ cytotoxic T lymphocyte differentiation
Source: JCI Insight. 2020 Feb 27;5(4):e133393. doi: 10.1172/jci.insight.133393 (PMC7101144; doi:10.1172/jci.insight.133393)
Supplement: Supplemental data [file jciinsight-5-133393-s081.pdf]

Supplementary Information

Histone deacetylase 1 and 2 restrain $CD4^+$ cytotoxic T lymphocyte differentiation

Authors: Teresa Preglej[1], Patricia Hamminger[1], Maik Luu[2], Tanja Bulat[3], Liisa Andersen[1], Lisa Göschl[1,4], Valentina Stolz[1], Ramona Rica[1], Lisa Sandner [1], Darina Waltenberger[1], Roland Tschismarov[5], Thomas Faux[6], Thorina Boenke[7], Asta Laiho[6], Laura L. Elo[6], Shinya Sakaguchi[1], Günter Steiner[4,8], Thomas Decker[5], Barbara Bohle[9], Alexander Visekruna[2], Christoph Bock[7,10], Birgit Strobl[4], Christian Seiser[11], Nicole Boucheron[1] and Wilfried Ellmeier[1]

[1]Division of Immunobiology, Institute of Immunology, Center for Pathophysiology, Infectiology and Immunology, Medical University of Vienna, Austria.

[2]Institute for Medical Microbiology and Hygiene, Philipps-University Marburg, Marburg, Germany.

[3]Institute of Animal Breeding and Genetics, Department of Biomedical Science, University of Veterinary Medicine Vienna, 1210 Vienna, Austria.

[4]Division of Rheumatology, Department of Internal Medicine III, Medical University of Vienna, 1090 Vienna, Austria.

[5]Max Perutz Labs, University of Vienna, Dr. Bohr-Gasse 9, 1030 Vienna

[6]Medical Bioinformatics Centre, Turku Bioscience Centre, University of Turku and Åbo Akademi University, Turku, Finland

[7]CeMM Research Center for Molecular Medicine of the Austrian Academy of Sciences, Vienna, Austria.

[8]Ludwig Boltzmann Institute for Arthritis and Rehabilitation, Vienna, Austria

[9]Department of Pathophysiology and Allergy Research, Center for Pathophysiology, Infectiology and Immunology, Medical University of Vienna, Vienna, Austria.

[10]Department of Laboratory Medicine, Medical University of Vienna, Vienna, Austria

[11]Division of Cell and Developmental Biology, Center for Anatomy and Cell Biology, Medical University of Vienna, Vienna, Austria.

Correspondence should be addressed to:

Wilfried Ellmeier, Division of Immunobiology, Institute of Immunology, Center for Pathophysiology, Infectiology and Immunology, Medical University of Vienna, Lazarettgasse 19, 1090 Vienna, Austria. Phone: +4314016033293; e.mail: wilfried.ellmeier@meduniwien.ac.at

Supplementary Figures

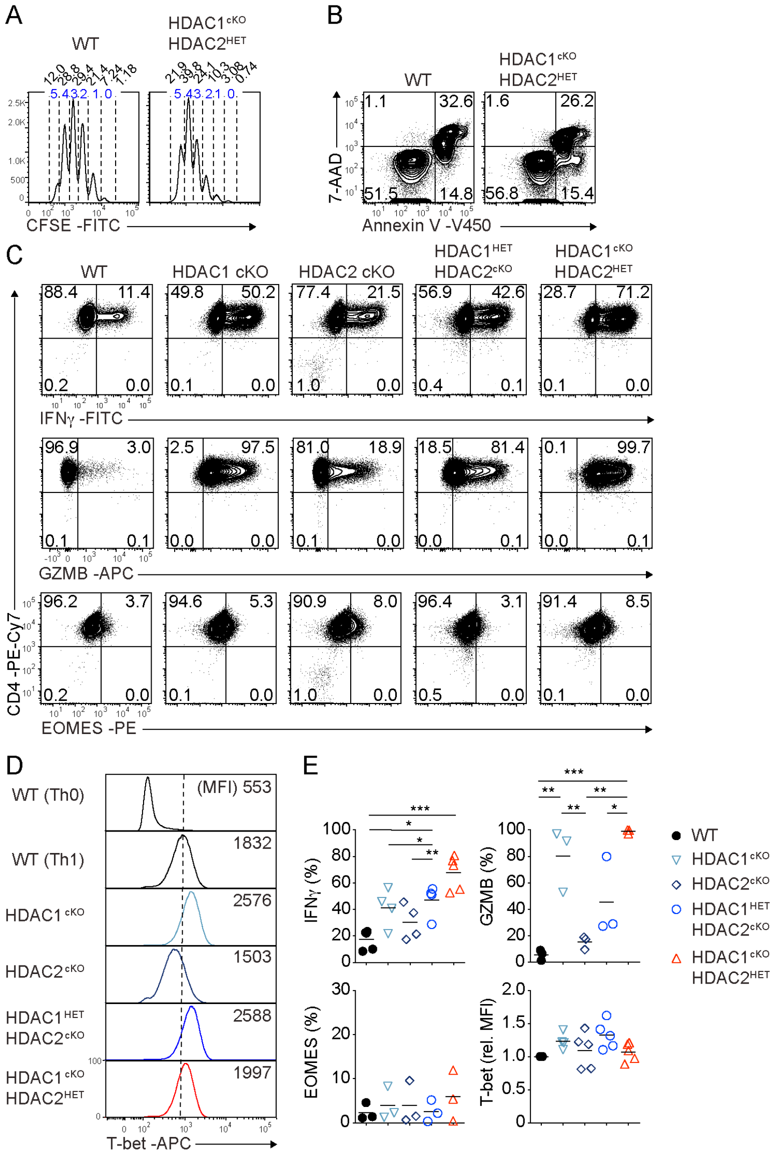

Figure S1. HDAC1/HDAC2 dosage-dependent upregulation of cytotoxic features in CD4$^+$ T cells. (A) Flow cytometry analysis depicting cell divisions of proliferation-dye-labeled WT and HDAC1^{cKO} -HDAC2^{HET} naïve CD4$^+$ T cells activated with anti-CD3/anti-CD28 for 3 days in the presence of IL-2. Numbers in gates indicate cell division cycle, whereas upper numbers indicate the percentage of cells in the respective cell division cycle gate. (B) 7AAD/AnnexinV staining of WT and HDAC1^{cKO}-HDAC2^{HET} CD4$^+$ T cells activated as described in A. (C) Flow cytometry analysis showing CD4, IFNγ, granzyme B (GZMB) and EOMES expression in WT, HDAC1^{cKO}, HDAC2^{cKO}, HDAC1^{HET}-HDAC2^{cKO} and HDAC1^{cKO}-HDAC2^{HET} naïve CD4$^+$ T cells activated with anti-CD3/anti-CD28 for 3 days in the presence of IL-2 under Th1 polarizing conditions (IL-12 and anti-IL-4). (D) Histograms depict T-bet expression in WT, HDAC1^{cKO}, HDAC2^{cKO}, HDAC1^{HET} -HDAC2^{cKO} and HDAC1^{cKO}-HDAC2^{HET} naïve CD4$^+$ T cells activated as described in C. (E) Summary diagrams showing the percentages (%) of cells of the indicated genotype expressing IFNγ, T-bet and EOMES as described in C. Each symbol indicates one mouse. Horizontal bars indicate the mean. *P<0.05, **P<0.01, ***P<0.001 (One-way ANOVA analysis followed by Tukey's multiple-comparison test). (A,B,C) Numbers indicate the mean fluorescence intensity (MFI) (A) or the percentage of cells in the respective quadrants or gates (B,C). (D) The dotted vertical lines indicate the peak of the WT histogram (for MFI). Data are representative (A,B,C,D) or show summary (E) of at least 2 (A,B), 5 (C,D,E) mice that were analyzed in at least 3 (A), 2 (B,C) and 4 (D,E) independent experiments.

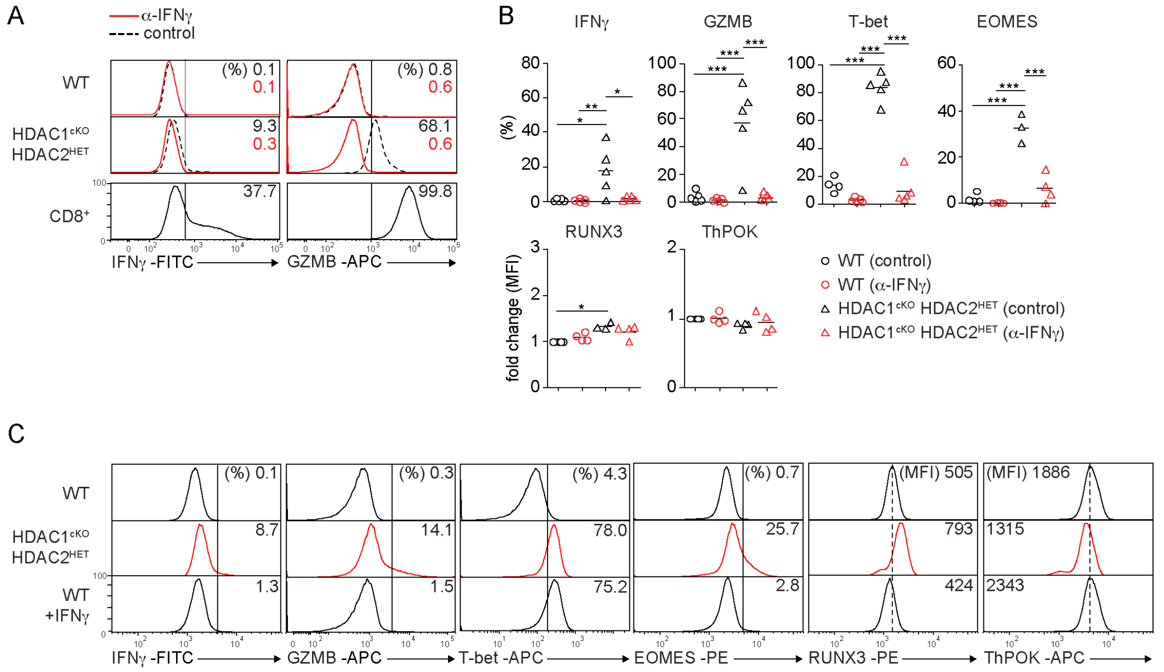

Figure S2. IFNγ signaling is necessary for the upregulation of cytotoxic features in HDAC1^{cKO}-HDAC2^{HET} mice. (A) Histograms depict IFNγ and granzyme B (GZMB) expression in naïve WT, HDAC1^{cKO}-HDAC2^{HET} CD4^{+} and WT CD8^{+} T cells activated with anti-CD3/anti-CD28 for 3 days in the presence or absence (control) of IFNγ blocking antibodies (α-IFNγ) and analyzed by flow cytometry. (B) Summary of experiments described in A. Diagrams depict ether the percentages (%) of CD4^{+} T cells expressing the indicated cytokines/transcription factors; or WT (control) MFI levels were set as 1 and relative MFI levels in WT (α-IFNγ) and HDAC1^{cKO}-HDAC2^{HET} (control/α-IFNγ) CD4^{+} T cells are shown. Each symbol indicates one independent biological sample. Horizontal bars indicate the mean. *$P<0.05$, **$P<0.01$, ***$P<0.001$ (One-way ANOVA analysis followed by Tukey's multiple-comparison test). (C) Histograms showing IFNγ, granzyme B (GZMB), T-bet, EOMES, RUNX3 and ThPOK expression in naïve WT, HDAC1^{cKO}-HDAC2^{HET} CD4^{+} and WT CD8^{+} T cells activated with anti-CD3/anti-CD28 for 3 days in the presence or absence of exogenous IFNγ and analyzed by flow cytometry. (A,C) Numbers indicate the percentage of cells in the respective quadrants or gates or as indicated mean fluorescence intensity (MFI). (A,C) The dotted vertical lines indicate the peak of the WT histogram (for MFI), while the vertical solid lines indicates the gating region for the percentage of cells. Data are representative of at least 5 (A-C) mice that were analyzed in at least 3 (A,B) and 2 (C) independent experiments.

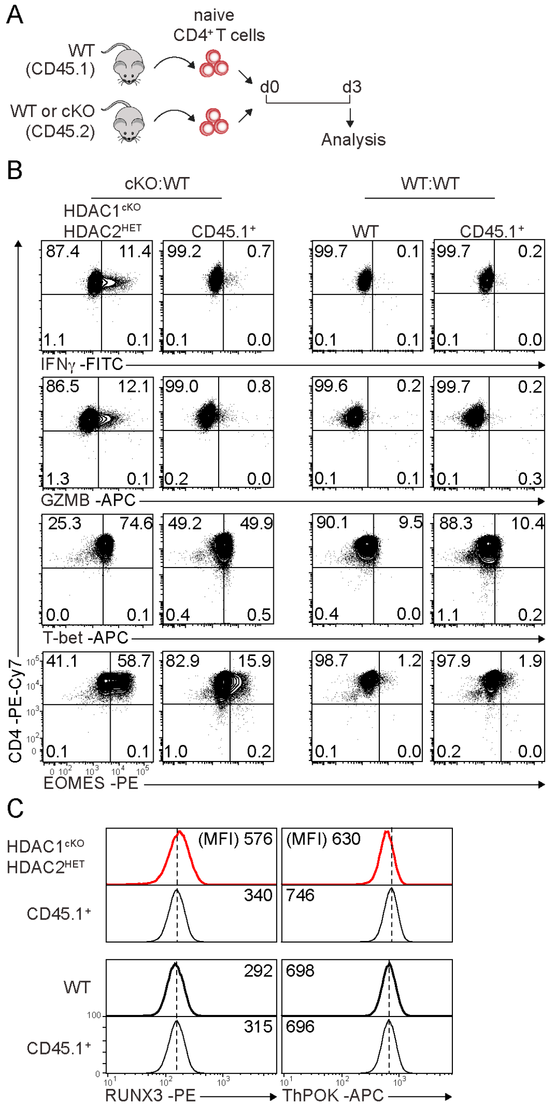

Figure S3. Upregulation of CD4+ CTL features in HDAC1cKO-HDAC2HET CD4+ T cells is induced by cell-intrinsic features. (A) Experimental outline for co-culture experiments: sorted naïve CD4+ T cells from either WT or HDAC1cKO-HDAC2HET mice (CD45.2+) and from congenic (CD45.1+) mice were mixed at a 1:1 ratio and activated with anti-CD3/anti-CD28 for 3 days. (B) Flow cytometry analysis showing IFNγ, granzyme B (GZMB), T-bet and EOMES expression in co-cultured HDAC1cKO-HDAC2HET and CD45.1+ CD4+ T cells, as well as WT and CD45.1+ CD4+ T cells. (C) Histograms depict RUNX3 and ThPOK expression in co-cultured HDAC1cKO-HDAC2HET/WT and CD45.1+ CD4+ T cells. (B,C) Numbers indicate the percentage of cells in the respective quadrants and gates or, as indicated mean fluorescence intensity (MFI). (C) The dotted vertical lines indicate the peak of the WT histogram (for MFI). Data are representative of at least 5 (B,C) mice that were analyzed in at least 3 (B,C) independent experiments.

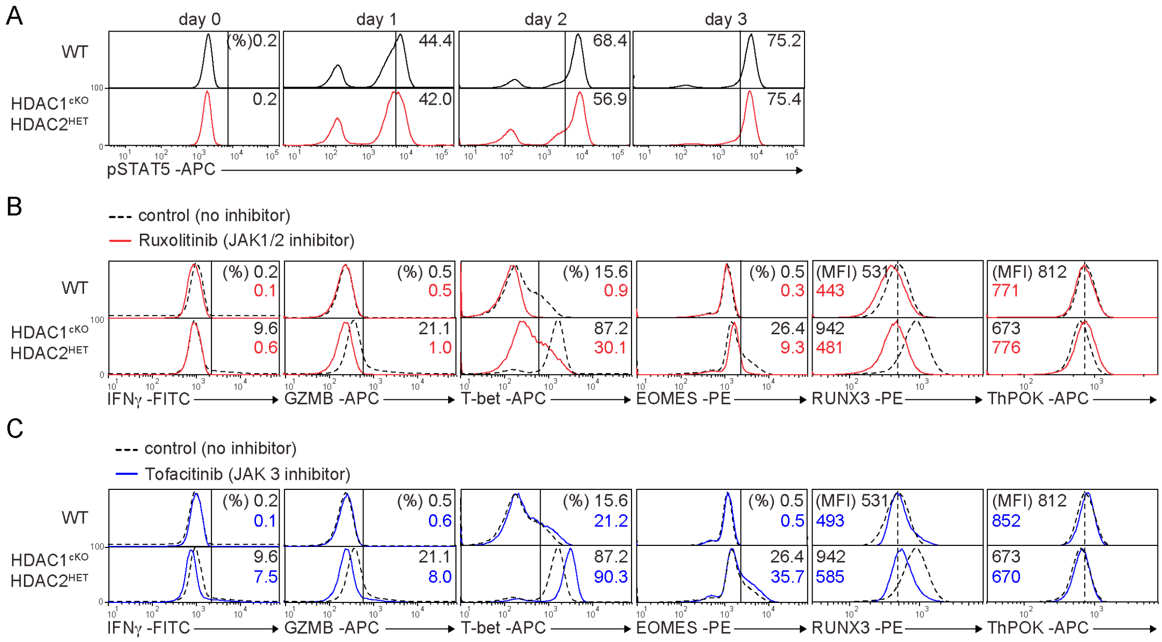

Figure S4. JAK1/JAK2 signaling is required for the upregulation of $CD4^+$ CTL features in $HDAC1^{cKO}$-$HDAC2^{HET}$ $CD4^+$ T cells. (A) Histograms depict pSTAT5 levels in naïve WT and $HDAC1^{cKO}$-$HDAC2^{HET}$ $CD4^+$ T cells activated with anti-CD3/anti-CD28 in the presence of IL-2 and analyzed by flow cytometry at the indicated time points. (B,C) Histograms showing IFNγ, granzyme B (GZMB), T-bet, EOMES, RUNX3 and ThPOK expression in naïve WT and $HDAC1^{cKO}$-$HDAC2^{HET}$ $CD4^+$ T cells activated with anti-CD3/anti-CD28 for 3 days in the presence or absence of the JAK1/2 inhibitor Ruxolitinib (B) or the JAK3 inhibitor Tofacitinib (C). (B,C) Dotted lines represent control cells (no inhibitor treatment). The same non-treated WT and $HDAC1^{cKO}$-$HDAC2^{HET}$ samples are shown in B and C. (A-C) Numbers indicate the percentage of cells in the respective regions or the mean fluorescence intensity (MFI). (A-C) The dotted vertical lines indicate the peak of the WT histogram (for MFI), while the vertical solid lines indicate the gating region for the percentage of cells. Data are representative of at least 4 (A-C) mice that were analyzed in at least 2 (A) and 4 (B,C) independent experiments.

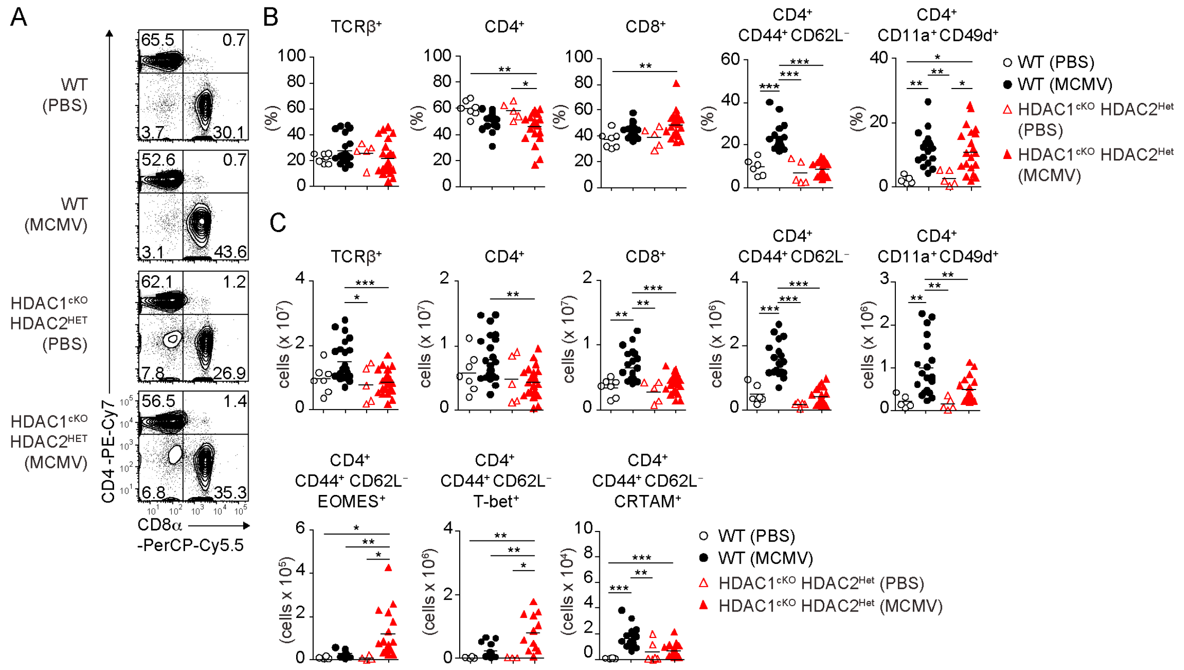

Figure S5. CD4^{+} CTL features are induced in HDAC1^{cKO}-HDAC2^{HET} CD4^{+} T cells upon MCMV infection. (A) Flow cytometry analysis of splenocytes isolated from WT and HDAC1^{cKO}-HDAC2^{HET} mice upon MCMV infection or control PBS injection showing CD4 and CD8 expression on viable TCRβ^{+} splenocytes. Numbers indicate the percentage of cells in the respective quadrants. (B,C) Diagrams show the percentage (B) and the total cell number (C) of TCRβ^{+}, CD4^{+}, CD8^{+}, CD4^{+} CD44^{+}CD62L^{-}, CD4^{+} CD11a^{+}CD49d^{+} viable splenocytes as well as of EOMES^{+}, T-bet^{+} and CRTAM^{+} CD4^{+}CD44^{+}CD62L^{-} viable splenocytes, respectively, isolated from WT and HDAC1^{cKO}-HDAC2^{HET} (MCMV and PBS-control) mice. Each symbol indicates one mouse. Horizontal bars indicate the mean. *P<0.05, **P<0.01, ***P<0.001 (One-way ANOVA analysis followed by Tukey's multiple-comparison test). Data are representative (A) or show a summary (B,C) of at least 16 infected (A-C) and 4-6 PBS injected (A-C) mice that were analyzed in at least 4 (A-C) independent experiments.

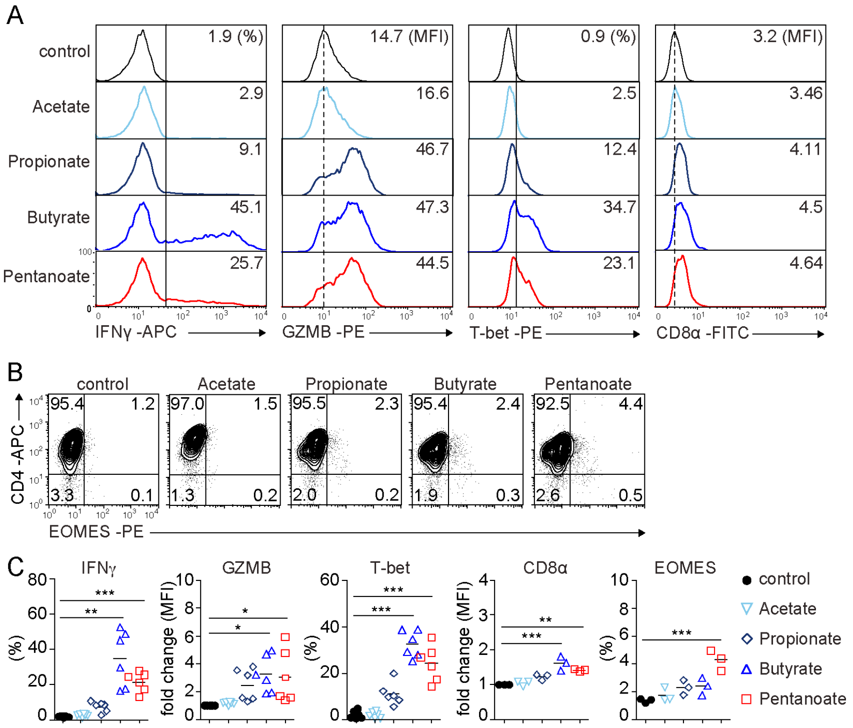

Figure S6. Murine CD4$^+$ T cells upregulate cytotoxic features upon SCFA treatment. (A) Histogram panels depict IFNγ, granzyme B (GZMB), T-bet and CD8α expression on anti-CD3/anti-CD28 activated murine CD4$^+$ T cells in the absence (control) or presence of acetate, propionate, butyrate and pentanoate, respectively, analyzed by flow cytometry (B) Flow cytometry analysis showing EOMES expression on murine CD4$^+$ T cells treated as described in (A). (C) Summary of experiments described in A. Diagrams depict ether the percentages (%) of CD4$^+$ T cells expressing the indicated cytokines/transcription factors; or control MFI levels were set as 1 and relative MFI levels in acetate-, propionate-, butyrate- or pentanoate-treated CD4$^+$ T cells are shown. Each symbol indicates one independent biological sample. Horizontal bars indicate the mean. *P<0.05, **P<0.01, ***P<0.001 (One-way ANOVA analysis followed by Tukey's multiple-comparison test). Data are representative (A,B) or show a summary (C) of 6 (A-C) mice that were analyzed in 2 (A-C) independent experiments.

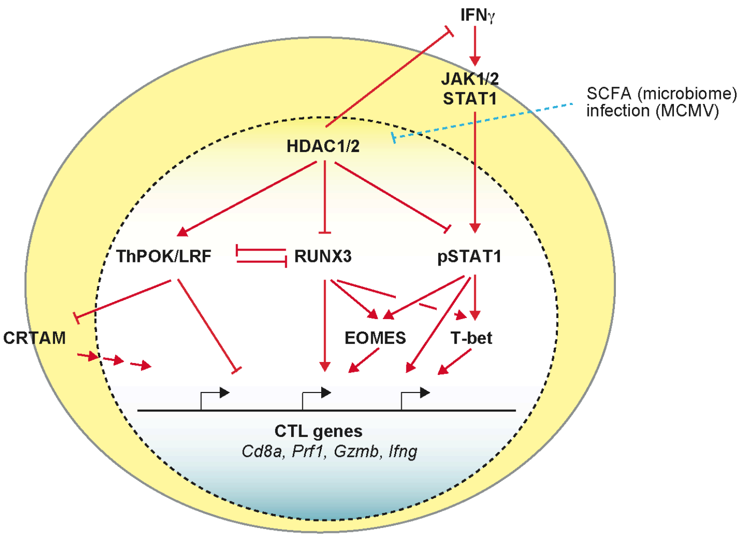

Figure S7. Model of how HDAC1/2 control CD4$^+$ CTL induction

Model depicting the role of HDAC1 and HDAC2 in restraining CD4$^+$ CTL differentiation. Adapted from Ref. #15. See discussion section for details.

Supplementary Table S1

List of genes preferentially expressed either in WT Th1 cells (“Th1-selective”) or in activated CD8$^+$ T cells ("activated CD8$^+$ T-selective”). Differential expression was defined as FC >_2; FDR ≤ 0.05 (Th1 and CD8$^+$ expression data were taken from Ref.#19).

**"Th1-selective" gene set (169)**	
2210010N04Rik	Olfr672
2610019F03Rik	Ormdl3
4933426M11Rik	P2rx7
5830405N20Rik	Pde2a
A530021J07Rik	Pip5k1b
A630052C17Rik	Ppp1r3f
Abtb1	Pros1
Actn1	Ptgir
Adam23	Rab37
Adamtsl4	Rasa3
Adh1	Rgs10
Aff3	Rnf138
AI747699	Rnf32
Aldh3b2	Rundc3a
Amigo2	Rxra
Arhgap15	S1pr1
Arhgef18	Scml4
Art2b	Sdcbp2
Asap1	Sell
Atn1	Sema3b
Atp10d	Sesn3
Axin2	Sh3bp5
B3galt2	Si
B630019A10Rik	Sidt1
Bach2	Slamf6
Bclp2	Slc14a1
Bmp7	Slc25a45
Btbd11	Slc28a2
Btla	Slfn1
Ccr7	Slfn2
Cd200r1	Smad3
Cd4	Snx33
Cd40lg	Sorcs2
Cd5	Spo11
Cd84	St3gal1
Cd9	St6gal1
Cd97	St8sia1
Chd3	St8sia6
Clec2d	Tas2r136
Crebl2	Tcf7
Crlf3	Tcp11l2
Csf2	Timp2
Cyp4f16	Tlr1
Dgkd	Tmem63a
Dst	Tmem64
Ecm1	Tmem71
EG218444	Tmprss13
EG240327	Tnfaip8l2
Ehd3	Tnfrsf25
ENSMUSG00000073540	Tnfrsf26
Fam134c	Tnfsf8
Fam169b	Trat1
Fam49a	Trib2
Fam53b	Trim12
Fcgr3	Trim34
Folr4	Tspan32
Foxp3	Ttc39b
Galnt10	Ttyh3

**"activated CD8$^+$ T-selective" gene set (477)**				
1110031I02Rik	Cercam	Gzmk	Nt5dc2	Sema7a
1500010J02Rik	Cfdp1	H2-Ab1	Nt5e	Sep11
2010002N04Rik	Chaf1b	H2-Oa	Nubpl	Setd8
2310016C08Rik	Chek1	Haao	Nudt5	Sfxn1
2310046K01Rik	Chst12	Hat1	Nudt9	Sgol2
2610028A01Rik	Cltb	Havcr2	Nuf2	Shmt2
2610318N02Rik	Clybl	Hells	Nup43	Sigmar1
2810417H13Rik	Cmtm7	Hk2	Nup93	Slamf7
2810452K22Rik	Cndp2	Hmgcr	Orc6l	Slc16a1
2900010J23Rik	Cnih4	Hmgcs1	Osbpl3	Slc19a1
2900010M23Rik	Coro2a	Hmmr	P2rx3	Slc1a4
2900026A02Rik	Creg1	Hook1	Pa2g4	Slc25a1
2900064A13Rik	Crybg3	Hprt1	Pafah1b3	Slc25a17
3000004C01Rik	Csda	Hpse	Park7	Slc3a2
4930572J05Rik	Cse1l	Hsd17b10	Pdcd1	Slc43a3
5730469M10Rik	Ctdspl2	Hsd17b7	Pdk3	Slc7a1
Aaas	Cuedc2	Hspa9	Pfkp	Slc7a3
Aacs	Cyb5r3	Hspb6	Phb	Slc7a5
Aars	Cyp51	Iars	Phf10	Smc2
Abcb1b	D5Wsu178e	Idh1	Phf16	Smpd1
Abce1	D930014E17Rik	Ier3	Pik3ap1	Sms
Acaca	D930046H04Rik	Ifi30	Pik3cg	Smyd2
Aco2	Dad1	Ikzf2	Pitrm1	Snai3
Acot11	Dars	Il10	Plcg2	Soat2
Actl6a	Dbi	Il12rb1	Plek	Spag5
Adam8	Dctd	Ilf2	Plekhf1	Spats2
Adssl1	Ddx1	Impa2	Plk1	Spcs1
Afg3l2	Dennd4a	Ipo5	Plod2	Spire1
AI847670	Dhcr24	Irf4	Pmvk	Sqle
Aim2	Dhfr	Irf8	Pnpt1	Srm
Ak3l1	Dhrs11	Isoc1	Pola1	St6galnac6
Alad	Dis3	Jup	Pola2	Stil
Aldh18a1	Dkkl1	Kcnk5	Pold2	Stra13
Aldh7a1	Dnm1	Kif15	Pole	Sult2b1
Aldh9a1	Dnmt1	Kif18a	Pole2	Supt3h
Aldoc	Dtl	Kif20a	Polr2d	Suv39h1
Alg9	E2f1	Klrc1	Polr2e	Syce2
Amica1	E2f8	Klrd1	Polr2f	Tanc1
Anln	Echdc1	Klrk1	Pop4	Tbc1d7
Anxa4	Eea1	Kpna3	Pp11r	Tcf19
Arhgap21	Eef2k	Lag3	Ppa1	Tec
Arhgdig	Ehd1	Lamc1	Ppap2c	Tfb1m
Asah1	Ehd4	Lap3	Ppil5	Tfg
Asf1b	Eif4ebp1	Larp1	Ppp1r3b	Tfrc
Asns	Emilin2	Lars	Prcp	Thoc3
Atp5d	Emp3	Lass6	Prdx5	Timm8b
Atpbd4	Eng	Ldlr	Prep	Tipin
Atpif1	Epcam	Litaf	Prf1	Tk1
Aurka	Ergic1	Lman1	Prim1	Tmem48
Aurkb	Erlin1	Lonp1	Prim2	Tmem97
B4galnt4	Ero1l	Lrp8	Prkar2a	Tmpo
Bag2	Esm1	Lrrk1	Prr11	Tnfrsf8
Banf1	Etfb	Lsm12	Prr5	Tnfrsf9
Bard1	Exo1	Lsm3	Prrg4	Tnfsf10
BC024814	Exosc8	Lsm4	Psat1	Topbp1
BC030867	Ezh2	Lss	Ptprj	Tpx2
Bcat1	F630043A04Rik	Lzts1	Ptprs	Trim13
Blm	Fads1	Mad2l1	Pus7	Trim37

Gata3	Utrn
Gbp2	Vav3
Gja1	Vipr1
Gjd3	Yes1
Gpm6b	Ypel3
Gpnmb	Ypel4
Gpr6	Zbtb7b
Gpr83	Zfp281
Gprc5b	Zfp36l1
Gramd3	Zhx2
Hs3st3b1	
Ifi27	
Ifit2	
Igfbp4	
Inpp4b	
Itga2	
Itgb3	
Itih5	
Itm2a	
Jub	
Kat2b	
Kctd12	
Klf2	
Klf3	
Klk1b9	
Lgmn	
Ly6i	
Lysmd1	
Lyst	
Map3k5	
Mrfap1	
Ms4a4b	
Ms4a4c	
Ms4a6b	
Ms4a6c	
Nanos2	
Nipa1	
Nsg2	
Olfr1036	
Olfr1362	
Olfr1396	
Olfr609	

Bnip3	Fads2	Mad2l2	Pycr1	Tspan31
Brca1	Fah	Mapk6	Qdpr	Ttc35
Brca2	Fam54a	Mars	Rab33a	Ttc39c
Bri3bp	Fam73a	Mastl	Rad51	Tubb6
Brip1	Fam96a	Mbnl3	Rad51ap1	Tubg1
Bsg	Fanci	Mcm10	Rad54l	Uhrf1
Bst1	Fasn	Mcm2	Rala	Usp10
Bub1	Fbxl5	Mcm5	Ran	Usp11
C1qbp	Fchsd2	Mcm6	Rars	Vat1
C530044N13Rik	Fdft1	Mcm7	Rbbp7	Vegfa
Cables1	Fh1	Megf9	Rbpj	Vegfb
Cad	Fignl1	Mfsd2	Rcn3	Vps29
Car12	Fkbp3	Mlkl	Rdbp	Wdhd1
Cars	Fndc3a	Morn2	Rdh11	Wdr67
Casc4	Foxm1	Mrpl19	Renbp	Wee1
Cbx5	Frrs1	Mrpl40	Rfc1	Yars
Ccdc101	Fzd7	Mrrf	Rfc3	Zbtb32
Ccl3	G6pc3	Msh2	Rfc4	Zdhhc8
Ccl5	Galc	Mt1	Rfc5	Zwilch
Ccna2	Galnt3	Mt2	Rhbdf2	
Ccnb2	Gatm	Mthfd1	Ripk3	
Ccne2	Gbe1	Mthfd1l	Rmnd5b	
Cd160	Gclm	Mthfd2	Rora	
Cd24a	Gem	Mvk	Rpl23	
Cd38	Gen1	Mybl1	Rqcd1	
Cd68	Gins1	Myo5a	Rras	
Cd72	Gins2	Nampt	Rrm1	
Cd79b	Gm996	Nars	Rrp1b	
Cd80	Gmds	Ncaph	Rtcd1	
Cd8a	Gnb5	Ndufs7	Ruvbl2	
Cd8b1	Gng12	Ndufs8	S100a1	
Cd93	Gnptab	Ndufv1	Sap30	
Cdc45l	Gpd2	Nedd4	Sar1b	
Cdc6	Gpr141	Nek1	Sass6	
Cdca7	Gpr65	Nkg7	Sc4mol	
Cdca8	Gpr68	Nmt2	Scarb1	
Cdh1	Gpr89	Nop16	Sccpdh	
Cdk4	Gpsn2	Nr4a2	Scd1	
Cdk6	Gtse1	Nrbp2	Scd2	
Cdkn1a	Gzma	Nsbp1	Sdf2l1	
Cenpn	Gzmb	Nsdhl	Selenbp1	
Cenpv	Gzmc	Nsl1	Selenbp2	

Supplementary Table S2

List of gene sets from the Molecular Signature Database that are up- (negative normalized enrichment score (NES)) or downregulated (positive NES) in activated HDAC1^{cKO}/HDAC2^{HET} CD4^{+} T cells. Gene sets in red fonts have been generated from a comparison of published Th1 and activated CD8^{+} T cell (CTL) microarray datasets (see methods section for details).

PATHWAY - UPREGULATED	NES
"ACTIVATED CD8^{+} T-SELECTIVE"	-1,34
APICAL JUNCTION	-1,28
KRAS SIGNALING	-1,19
IL6-JAK-STAT3 SIGNALING	-1,15
UV RESPONSE UP	-1,14
IL2-STAT5 SIGNALING	-1,07
INTERFERON-GAMMA RESPONSE	-1,06
INFLAMMATORY RESPONSE	-1,06
EPITHELIAL MESENCHYMAL TRANSITION	-1,05
ADIPOGENESIS	-1,03
COMPLEMENT	-1,02
ALLOGRAFT REJECTION	-1,00
KRAS SIGNALING UP	-0,97
MYOGENESIS	-0,92
INTERFERON-ALPHA RESPONSE	-0,88
HYPOXIA	-0,86
GLYCOLYSIS	-0,82
APOPTOSIS	-0,80
P53 PATHWAY	-0,76
XENOBIOTIC METABOLISM	-0,74

PATHWAY - DOWNREGULATED	NES
"Th1-SELECTIVE"	1,44
MITOTIC SPINDLE	1,06
EARLY ESTROGEN RESPONSE	0,99
TNFA SIGNALING VIA NFKB	0,97
COAGULATION	0,95
LATE ESTROGEN RESPONSE	0,93
HEME METABOLISM	0,71
UV RESPONSE DOWN	0,66
